# Mosaic results after preimplantation genetic testing for aneuploidy may be accompanied by changes in global gene expression

**DOI:** 10.3389/fmolb.2023.1180689

**Published:** 2023-04-14

**Authors:** A. Martin, A. Mercader, F. Dominguez, A. Quiñonero, M. Perez, R. Gonzalez-Martin, A. Delgado, A. Mifsud, A. Pellicer, M. J. De Los Santos

**Affiliations:** ^1^ IVI-RMA Foundation, Health Research Institute La Fe, Valencia, Spain; ^2^ IVI-RMA Valencia, Valencia, Spain; ^3^ IVI-RMA Rome, Rome, Italy

**Keywords:** embryonic mosaicism, RNA sequencing, preimplantation genetic testing for aneuploidy, next-generation sequencing, blastomere cell cycle

## Abstract

Aneuploidy in preimplantation embryos is a major cause of human reproductive failure. Unlike uniformly aneuploid embryos, embryos diagnosed as diploid-aneuploid mosaics after preimplantation genetic testing for aneuploidy (PGT-A) can develop into healthy infants. However, the reason why these embryos achieve full reproductive competence needs further research. Current RNA sequencing techniques allow for the investigation of the human preimplantation transcriptome, providing new insights into the molecular mechanisms of embryo development. In this prospective study, using euploid embryo gene expression as a control, we compared the transcriptome profiles of inner cell mass and trophectoderm samples from blastocysts with different levels of chromosomal mosaicism. A total of 25 samples were analyzed from 14 blastocysts with previous PGT-A diagnosis, including five low-level mosaic embryos and four high-level mosaic embryos. Global gene expression profiles visualized in cluster heatmaps were correlated with the original PGT-A diagnosis. In addition, gene expression distance based on the number of differentially expressed genes increased with the mosaic level, compared to euploid controls. Pathways involving apoptosis, mitosis, protein degradation, metabolism, and mitochondrial energy production were among the most deregulated within mosaic embryos. Retrospective analysis of the duration of blastomere cell cycles in mosaic embryos revealed several mitotic delays compared to euploid controls, providing additional evidence of the mosaic status. Overall, these findings suggest that embryos with mosaic results are not simply a misdiagnosis by-product, but may also have a genuine molecular identity that is compatible with their reproductive potential.

## 1 Introduction

Preimplantation genetic testing for aneuploidy (PGT-A) enables the identification of embryos carrying chromosomal alterations after *in vitro* fertilization (IVF). Embryos with uniform aneuploidies, resulting from meiotic errors during gametogenesis, are deselected for transfer due to the high risk of adverse clinical outcomes (Capalbo et al., 2022). Intermediate chromosome copy-number values in trophectoderm (TE) biopsies are often interpreted as evidence of chromosomal mosaicism (Paulson and Treff, 2020). Embryos with putative diploid-aneuploid mosaicism, which originates from mitotic errors after fertilization, have the potential to develop into healthy offspring (McCoy, R. C., 2017; Treff and Marin, 2021; Greco et al., 2023). However, the clinical management of these embryos remains the subject of an intense debate.

Retrospective cohort studies involving large data sets of embryo transfers have suggested that mosaic embryos have a reduced developmental potential compared to their euploid peers, and that the level and type of mosaicism can determine reproductive outcomes (Abhari and Kawwass, 2021). However, prospective non-selection studies have shown that mosaic embryos with less than 50% of aneuploid cells in the TE biopsy have equivalent developmental potential compared to fully euploid ones (Capalbo et al., 2021; Tiegs et al., 2021; Wang et al., 2021).

This view is supported by several systematic reviews and meta-analyses that have used the Newcastle-Ottawa scale to assess the quality of non-randomized studies (Mourad et al., 2021; Ma et al., 2022). Importantly, these studies agree that transferring a mosaic embryo entails a low risk of confirming the abnormal karyotype in the ensuing pregnancy. In fact, reports on this matter are limited (Kahraman et al., 2020; Schlade-Bartusiak et al., 2022; Greco et al., 2023).

In line with the debate, a recent position statement issued by the PGDIS on the management of embryonic mosaicism indicates that the decision to transfer a mosaic embryo can be prioritized either based on the level of mosaicism or the type of mosaicism (Leigh et al., 2022). Conversely, the ESHRE Working Group on Chromosomal Mosaicism avoids formulating specific recommendations to the transfer of high-level mosaic embryos due to the absence of robust clinical data (De Rycke et al., 2022).

Two major hypotheses have been proposed to explain why mosaic embryo transfers are compatible with positive pregnancy outcomes and the delivery of healthy offspring.

The first hypothesis considers that mosaicism is overestimated by false positive diagnoses. This notion is primarily supported by studies involving embryo disaggregation, which have demonstrated low concordance rates of the mosaic result among the diagnostic TE biopsy, the inner cell mass (ICM) and/or the remaining TE cells (Popovic et al., 2020; Capalbo et al., 2021; Marin et al., 2021). Furthermore, even diagnostic platforms validated for detecting mosaicism may not fully account for the technical noise introduced by TE biopsies, which can potentially be misinterpreted as a biological signal, leading to false positive diagnoses of mosaicism (Treff and Marin, 2021).

The second hypothesis supports the ability of mosaic embryos to self-correct. Reversion of aneuploidy appears to be a rare phenomenon in human embryos, given the low frequency of uniparental disomy at the blastocyst stage (Gueye et al., 2014; Taylor et al., 2014). Rather, a more-supported view of self-correction may be the negative selection of aneuploid cells during embryonic development through mechanisms of cell competition, such as apoptosis or differential cell proliferation (Bolton et al., 2016; Baker, 2020; Singla et al., 2020; Coticchio et al., 2021).

Against this background, there is an overriding need to study the biological implications of mosaic findings on the specific functions of the preimplantation embryo. RNA-sequencing (RNA-seq) techniques can be used to measure relative changes in gene expression across multiple experimental conditions (Ura et al., 2022). Conveniently, the optimization of library preparation protocols for use with low input samples has paved the way for the study of the human preimplantation transcriptome (Tang, F. et al., 2009; Islam et al., 2011; Ramskold et al., 2012; Picelli et al., 2014; Hwang et al., 2018).

In view of recent findings suggesting that moderate levels of mosaicism in the TE biopsy are not detrimental for embryo development (Capalbo et al., 2022), it is paramount to investigate whether such mosaic findings are accompanied by changes in global gene expression, compared to embryos having fully euploid karyotypes. Here, we used RNA-seq to compare the transcriptome profiles of ICM and TE samples from embryos diagnosed as euploid, low-level mosaic and high-level mosaic after PGT-A. The identification of pathways specifically deregulated in embryos with different levels of putative mosaicism may shed new light on the mechanisms leading to their reproductive competence.

## 2 Material and methods

### 2.1 Ethical approval

The study was approved by the National Commission of Human Reproduction (CNRHA), the general direction of research, innovation, technology, and quality and by our institutional review board, the ethics committee of Clinical Research IVI-RMA Valencia (#1710-VLC-102-MD), which complies with Spanish law on assisted reproductive technologies (14/2006) and biomedical research (14/2007).

### 2.2 Study design and population

This prospective cohort study was conducted on a total of 14 blastocysts donated to research by 12 couples who underwent IVF treatment with intracytoplasmic sperm injection (ICSI) and PGT-A at IVI-RMA (Valencia, Spain). The study cohort included blastocysts classified as euploid (*n* = 5), low-level euploid-aneuploid mosaic (*n* = 5) and high-level euploid-aneuploid mosaic (*n* = 4) after PGT-A. All participants provided written informed consent.

The mean age of patients providing oocytes and sperm was 35.8 ± 5.3 and 36.5 ± 6.2 years, respectively. Indications for PGT-A included advanced female age (≥35 years) (41.7% = 5/12), implantation failure (25% = 3/12), male factor (8.3% = 1/12), previous chromosomopathy (8.3% = 1/12) and multiple indications (16.7% = 2/12).

### 2.3 Ovarian stimulation and *in vitro* fertilization

Controlled ovarian stimulation was performed using GnRH antagonist protocol, as described elsewhere (Labarta et al., 2018). Recombinant hCG (rhCG) (Ovitrelle, Merck Serono, Germany) or GnRH agonist (Decapeptyl, Ipsen Pharma, France) was administered to trigger ovulation, and transvaginal oocyte retrieval was performed 36 h after rhCG or GnRH agonist administration. Oocyte retrieval, denudation, and ICSI were performed according to standard clinical practice at Instituto Valenciano de Infertilidad (Alegre et al., 2019). Embryos were incubated in Gems culture medium (Genea Biomedx, Australia) under mineral oil at 37°C, 6% CO_2_, and 5% O_2_ (balanced with N_2_) up to the blastocyst stage.

### 2.4 Preimplantation genetic testing for aneuploidy

All embryos underwent assisted hatching on day 3 using laser technology (Fertilase^®^, Octax, Sweden). On days 5 or 6, embryo quality was assessed according to guidelines from the Spanish Association for the Study of Biology of Reproduction, with slight modifications (Ardoy et al., 2008). Embryos that developed beyond a full blastocyst (graded A, B, or C) were considered for biopsy, which was performed by the pulling method (Aoyama and Kato, 2020). After biopsy, blastocysts were vitrified by the Kitazato method (Kuwayama, 2007; Cobo et al., 2009).

PGT-A was performed by next-generation sequencing (NGS) (Igenomix, Spain). Library construction, DNA sequencing and bioinformatic analysis were performed using the Ion ReproSeq PGS kit, Ion Chef Instrument, Ion S5 System and Ion Reporter software (Thermo Fisher Scientific, United States). An internal algorithm validated by Igenomix was used for mosaicism calling (Garcia-Pascual et al., 2020). TE biopsies were classified as i) Euploid: <30% of aneuploid cells; ii) Low-level mosaic: 1–2 chromosomes aneuploid in 30%–<50% cells; and iii) High-level mosaic: 1–2 chromosomes aneuploid in 50%–<70% cells. Segmental and/or sex chromosomal mosaicism were not detected.

### 2.5 Collection of inner cell mass and trophectoderm samples

**FIGURE 1:**
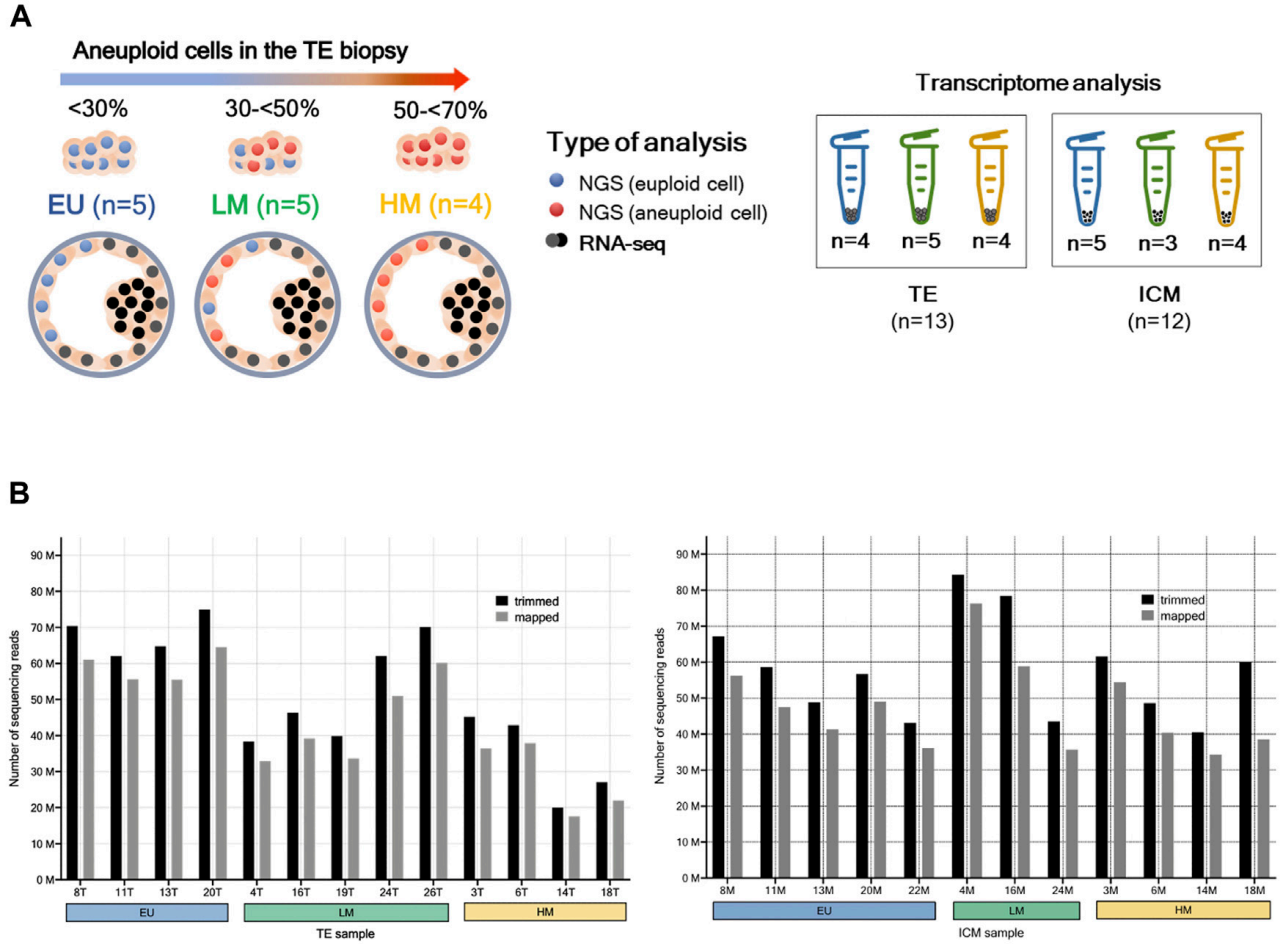
RNA-sequencing. **(A)** Study design. A single trophectoderm (TE) biopsy was analysed by next-generation sequencing (NGS). Blastocysts were classified as i) euploid (EU): <30% aneuploid cells, ii) low-level mosaic (LM): 30%-<50% aneuploid cells, and iii) high-level mosaic (HM): 50%–<70% aneuploid cells. The remaining TE (grey cells) and the inner cell mass (ICM) (black cells) were separately collected from each blastocyst and analysed by RNA sequencing (RNA-seq). **(B)** Transcriptome sequencing reads of TE and ICM samples. Trimmed reads were obtained through the elimination of adapter sequences and the removal of poor-quality bases from input reads. Mapped reads were aligned to the reference genome.

Blastocyst warming was performed by the Kitazato method (Kuwayama, 2007; Cobo et al., 2009). Following re-expansion, blastocysts were placed under an inverted microscope and held firmly with a holding pipette (SHP-120B-35 Sunlight Medical, United States). The ICM was identified as a group of cohesive, oval-shaped cells located inside the embryo. An ICM biopsy was performed by the pulling method, whereby cells were carefully drawn into a biopsy pipette (SBB-30Z-30 Sunlight Medical, United States) by applying gentle suction and 4–5 laser pulses of 3.1 milliseconds (Aoyama and Kato, 2020). The ICM biopsy and the remaining TE were separately collected from 14 blastocysts into RNAse-free PCR tubes containing 2 µL of 10X reaction buffer (SMART-Seq v4 Ultra-Low Input RNA kit for Sequencing, Takara Bio, United States), which were immediately stored at -80°C until processing (Figure 1A). Table 1 summarizes the main characteristics of the embryos included in the study.

**TABLE 1:** Study population and embryo characteristics. PGT-A was performed by next-generation sequencing (mosaic range defined between 30%–70% aneuploid cells). ^a^ Days post-fertilization. ^b^ Embryo quality assessed according to guidelines from the Spanish Association for the Study of Biology of Reproduction (ASEBIR).

Embryo number	Day of biopsy^a^	Grade^b^	NGS (% of aneuploid cells in the TE biopsy)	PGT-A diagnosis
EU1 (08)	5	B	(<30%)	Euploid
EU2 (11)	6	B	(<30%)	Euploid
EU3 (13)	5	C	(<30%)	Euploid
EU4 (20)	5	B	(<30%)	Euploid
EU5 (22)	5	B	(<30%)	Euploid
LM1 (04)	5	B	+6 (30%–50%), −7 (30%–50%)	Low-level mosaic
LM2 (16)	6	C	−22 (30%–50%)	Low-level mosaic
LM3 (19)	5	B	+4 (30%–50%)	Low-level mosaic
LM4 (24)	5	C	+8 (30%–50%), −22 (30%–50%)	Low-level mosaic
LM5 (26)	5	B	+12 (30%–50%), +20 (30%–50%)	Low-level mosaic
HM1 (03)	6	C	+4 (50%–70%)	High-level mosaic
HM2 (06)	5	B	+8 (50%–70%)	High-level mosaic
HM3 (14)	5	B	+19 (50%–70%)	High-level mosaic
HM4 (18)	5	C	−20 (50%–70%)	High-level mosaic

### 2.6 Library preparation and RNA sequencing

Total RNA was extracted from 28 samples using a SMART-Seq V4 Ultra-Low Input RNA Sequencing kit (Takara Bio, United States) that uses oligo (dT) priming. cDNA was synthesized with 3′SMART-Seq CDS primer II (Takara Bio, United States) and PCR-amplified (17 cycles) from 10 pg of RNA following manufacturer’s instructions. Amplified cDNA was purified using AMPure XP magnetic beads (Illumina, United States). After confirming cDNA integrity on a 2,100 Bioanalyzer (Agilent Technologies, United States), 1 ng of cDNA per sample were fragmented, and libraries were constructed using NexteraXT DNA sample preparation (Illumina, United States) according to manufacturer’s instructions. Samples were quantified using Qubit dsDNA Quantitation Assay (Thermo Fisher Scientific, United States), and three samples were excluded due to poor cDNA quality. An RNA pool was generated with 25 samples using equal concentration (5 nM) of RNA per sample. Barcodes and adapters were included in the library to sequence and identify all samples. Sequencing was performed in duplicate in a single run using an Illumina NovaSeq 6000 S1 platform (Illumina, United States) with a 200-nucleotide read length in a paired-end design (100-bp fragments). FastQC was used for checking the quality of the raw sequence data. Fragments that did not meet quality requirements were trimmed using Trimmomatic (Bolger et al., 2014). Alignment and quantification were performed using the Salmon algorithm (reference genome GRCh38) (Patro et al., 2017). An average of 54, 238, 594 reads were sequenced per sample, and 83.8% were successfully mapped (Figure 1B). Raw counts were directly used for differential gene expression analysis.

### 2.7 Bioinformatic analysis

Correlation studies, principal component analysis (PCA) and differential gene expression analysis were performed with the DESeq2 package (Love et al., 2014). Venn diagrams were generated with the software EVenn (Chen et al., 2021). Volcano plots were generated with the software VolcaNoseR (Goedhart and Luijsterburg, 2020). The fgsea algorithm was used for functional enrichment analysis on Kyoto Encyclopedia of Genes and Genomes (KEGG) pathways and Gene Ontology (GO) terms; i.e., Biological Process (GO-BP), Molecular Function (GO-MF) and Cellular Component (GO-CC) (Korotkevich et al., 2019). Enrichment scores (ES) were calculated as the degree to which each gene set was overrepresented at the top or bottom of the ranked list of genes in the expression dataset. Normalized enrichment scores (NES) were obtained after normalization across the analyzed gene sets. Gene set enrichment results were analyzed using the tool EnrichmentMap in Cytoscape (Shannon et al., 2003).

Six different comparisons were analyzed: (C-1) TE of euploid blastocysts (*n* = 4) vs. TE of low-level mosaic blastocysts (*n* = 5); (C-2) TE of euploid blastocysts (*n* = 4) vs. TE of high-level mosaic blastocysts (*n* = 4); (C-3) TE of low-level mosaic blastocysts (*n* = 5) vs. TE of high-level mosaic blastocysts (*n* = 4); (C-4) ICM of euploid blastocysts (*n* = 5) vs. ICM of low-level mosaic blastocysts (*n* = 3); (C-5) ICM of euploid blastocysts (*n* = 5) vs. ICM of high-level mosaic blastocysts (*n* = 4); and (C-6) ICM of low-level mosaic blastocysts (*n* = 3) vs. ICM of high-level mosaic blastocysts (*n* = 4).

### 2.8 Validation of RNA-seq by quantitative PCR

To corroborate gene expression data from RNA-seq, cDNA samples from each comparison group were analyzed by quantitative real-time PCR (qRT-PCR) on a StepOnePlus system (Applied Biosystems, United States) using Power-Up SYBR green (Thermo Fisher Scientific, United States). Duplicate cDNA amplifications were performed on the same cDNA samples used in RNA-seq (primer descriptions provided in Supplementary Table S1).

Six genes were randomly selected amongst the top differentially-expressed genes, based on their fold change, statistical significance and inclusion within significantly-enriched functions in the context of embryo development:

i) Rho GTPase Activating Protein 36 (*ARHGAP36*): FC = 8.1; padj = 1.5E-04 (C-1).
ii) Neural EGFL Like 1 (*NELL1*): FC = −7.9; padj = 1.8E-03 (C-1) and FC = −8.6; padj = 1.2E-06 (C-2).
iii) Neurotrophic Receptor Tyrosine Kinase 3 (*NTRK3*): FC = 5.5; padj = 2.9E-04 (C-4).
iv) Cadherin 5 (*CDH5*): FC = 8.2; padj = 6.6E-03 (C-5) and FC = 8.3; padj = 8.5E-03 (C-6).
v) Vimentin (*VIM*): FC = −8.8; padj = 2.1E-04 (C-5).
vi) Laminin Subunit Gamma 3 (*LAMC3*): FC = −4.5; padj = 1.2E-05 (C-6).

Selected genes are highlighted in Supplementary Figure S1. These genes were found in several GO annotation classes in order to avoid bias towards a particular function, and were related to embryonic cell growth, differentiation and survival (*ARHGAP36; NELL1; NTRK3; VIM*), as well as to cell migration, adhesion and organization of cells into tissues during embryogenesis (*CDH5; LAMC3*).

Gene expression levels were normalized to the housekeeping gene glyceraldehyde-3-phosphate dehydrogenase (*GAPDH*) and quantified by the ΔΔCT method. Fold change was calculated as the normalized gene expression (2∧ (−ΔCT)) in each sample divided by the normalized gene expression (2∧ (−ΔCT)) in a random reference sample.

### 2.9 Analysis of blastomere cell cycles by time lapse imaging

The development of low-level mosaic embryos (*n* = 4) and high-level mosaic embryos (*n* = 4) was retrospectively analyzed from the 1- to 8- cell stage by means of time lapse imaging (TLI). The timings of 8 direct morphokinetic variables (expressed in hours after ICSI) were annotated: extrusion of the second polar body (tPB2); division to 2, 3, 4, 5, 6, 7, and 8 cells (t2, t3, t4, t5, t6, t7, and t8). Duration of blastomere cell cycles were calculated as indirect morphokinetic variables (expressed in hours) as described by (Grau et al., 2015): duration of the first blastomere cell cycle (cc1 = t2-tPB2); duration of the second cell cycle of the first blastomere to cleave from the 2- to the 3-cell stages (cc2a = t3–t2); duration of the second cell cycle of the second blastomere to cleave from the 2- to the 4-cell stages (cc2b = t4-t2); mean cc2 (cc2m) as the mean of cc2a and cc2b; duration of the third cell cycle of the first (cc3a), second (cc3b), third (cc3c), and fourth (cc3d) blastomeres to cleave to the 5-, 6-, 7-, and 8-cell stages, respectively. Importantly, tracking blastomere origin was necessary to determine cc3a, cc3b, cc3c, and cc3d. Mean cc3 (cc3m) was calculated as the mean of cc3a, cc3b, cc3c, and cc3d. Since TLI videos were not available for the same euploid embryos used in the RNA-seq study, morphokinetic timings of putative mosaic embryos were compared to those from a random subset of 25 embryos of similar morphological quality which had been diagnosed as euploid with the same NGS protocol and analyzed in a previous study (Martin et al., 2021).

### 2.10 Statistical analysis

Differential gene expression analysis was performed using the parametric Wald test with Benjamini–Hochberg adjustment (padj). Genes with padj <0.05 and a log2FoldChange (FC) of ±2 were considered significantly differentially expressed (DEGs). GO terms and KEGG pathways with padj<0.05 were considered significantly deregulated. Validation analyses were performed by using a two-tailed *t*-test for independent samples, where *p* < 0.05 was considered statistically significant. Data analysis and figure preparation were performed in R and GraphPad Prism 9 (GraphPad Software, United States). Morphokinetic timings were compared by one-way ANOVA and Bonferroni *post hoc*, and variables were expressed as mean ± standard deviation.

## 3 Results

### 3.1 Comparison of global gene expression profiles

We first analyzed the differences in global gene expression across the six comparisons using PCA and cluster heatmaps. In the PCA, we observed that the dominant expression patterns captured by the first two principal components were not related to the original PGT-A result. Samples were only minimally separated by the PGT-A diagnosis when comparing euploid embryos to high-level mosaic embryos, both in TE comparisons and in ICM comparisons (Supplementary Figure S2). In contrast, in the cluster heatmaps, samples were grouped according to the PGT-A diagnosis in all comparisons, and expression vectors for samples within the same cluster were much more similar than those for samples from different clusters (Supplementary Figure S3). Only one TE sample collected from a low-level mosaic embryo was clustered in a different group (Supplementary Figure S3A). Notably, the TE samples of low-level mosaic embryos and high-level mosaic embryos showed the greatest degree of similarity (Supplementary Figure S3C).

### 3.2 Analysis of differentially expressed genes

**FIGURE 2:**
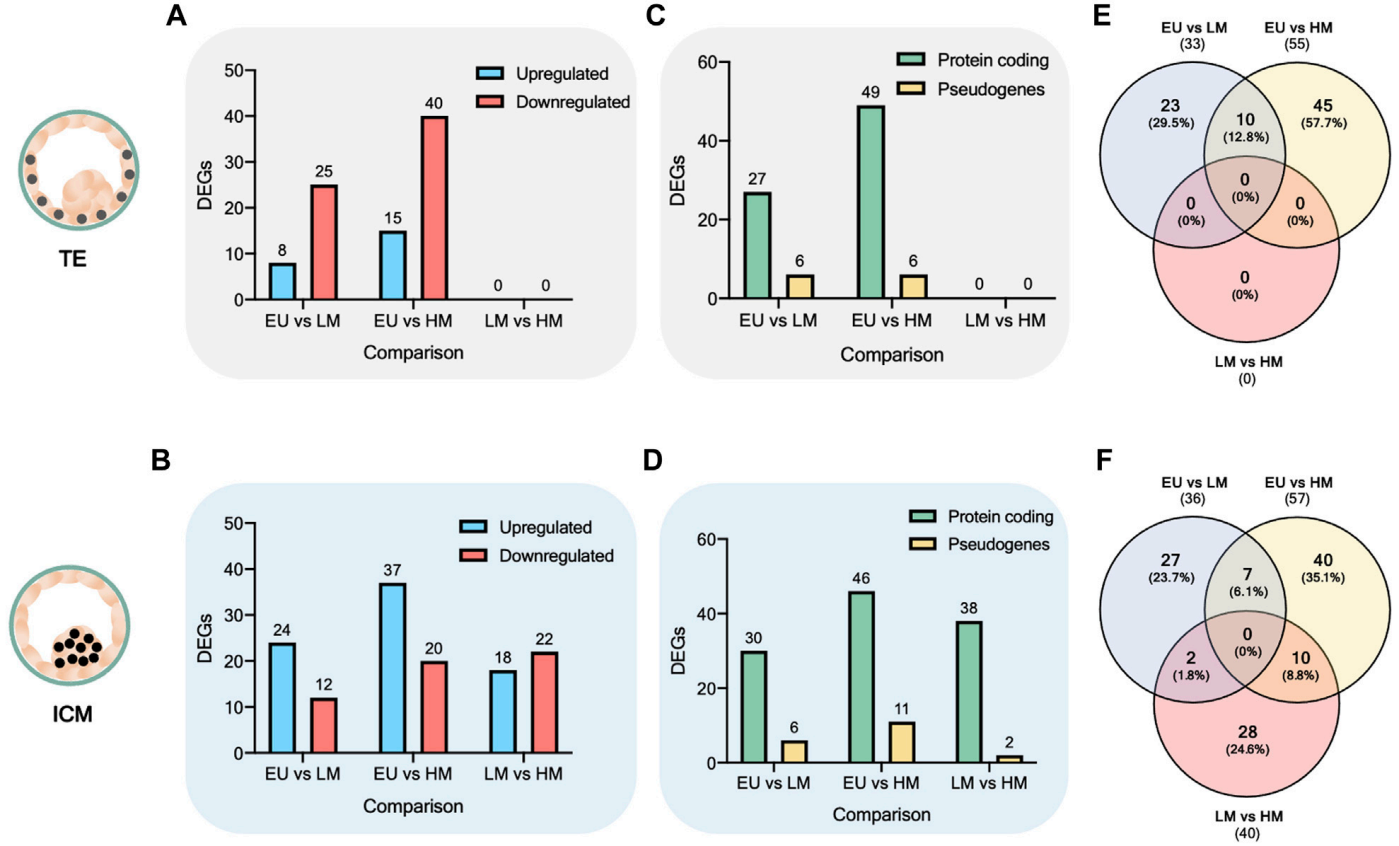
Analysis of differentially expressed genes. **(A–B)** Number of differentially expressed genes (DEGs). DEGs are upregulated (blue) or downregulated (red) in the first comparison factor. **(C–D)** Biotype of DEGs. **(E–F)** Venn diagram of overlap between DEGs.

Next, we identified the statistically significant DEGs across the six comparisons. We observed that the number of DEGs increased in both the TE (Figure 2A) and the ICM (Figure 2B) when comparing euploid embryos to low-level mosaic embryos (33 DEGs in the TE and 36 DEGs in the ICM) and high-level mosaic embryos (55 DEGs in the TE and 57 DEGs in the ICM). However, no statistically significant DEGs were found between the TE of low-level mosaic embryos and high-level mosaic embryos, indicating that the differences observed between both groups were not statistically significant. In contrast, 40 DEGs were found between the ICM samples of low-level and high-level mosaic embryos. A volcano plot was used to represent the differential expression of genes between groups (Supplementary Figure S1).

Most of the DEGs found were protein coding, accounting for 76 out of 88 (86.4%) DEGs in TE comparisons (Figure 2C) and 114 out of 133 (85.7%) DEGs in ICM comparisons (Figure 2D), while the rest were categorized as pseudogenes. The full list of DEGs is provided in Supplementary Table S2.

Next, we used the Venn diagram to identify overlapping and unique statistically significant DEGs. A total of 17 DEGs were found commonly significantly deregulated (upregulated or downregulated) when comparing euploid embryos to both low-level mosaic embryos and high-level mosaic embryos, respectively (Supplementary Table S3). 10 out of the 17 DEGs were deregulated in the TE (Figure 2E), and 7 DEGs were deregulated in the ICM (Figure 2F).

In addition, 10 DEGs were found commonly significantly deregulated in the ICM of high-level mosaic embryos, compared to the ICM of both euploid and low-level mosaic embryos (Supplementary Table S4). Finally, a total of 40 DEGs were found exclusively significantly deregulated in the ICM of high-level mosaic embryos compared to euploid embryos (Supplementary Table S5).

### 3.3 Functional enrichment analysis

#### 3.3.1 Trophectoderm cells

Since the TE cells of low-level mosaic embryos and high-level mosaic embryos had similar gene expression profiles (0 DEGs), we expected to find commonly deregulated pathways compared to euploid embryos. Indeed, gene set enrichment analysis revealed 124 terms that were commonly significantly deregulated in the TE of both low-level mosaic embryos and high-level mosaic embryos, compared to euploid controls. 85 out of the 124 (68.6%) deregulated terms were GO-Biological Process (GO-BP), 27 (21.8%) were GO-Cellular Component (GO-CC), 7 (5.6%) were GO-Molecular Function (GO-MF), and 5 (4%) were KEGG pathways. These included chromosome segregation, ERAD pathway, regulation of apoptotic signaling pathway, and steroid biosynthesis (Supplementary Table S6).

**FIGURE 3:**
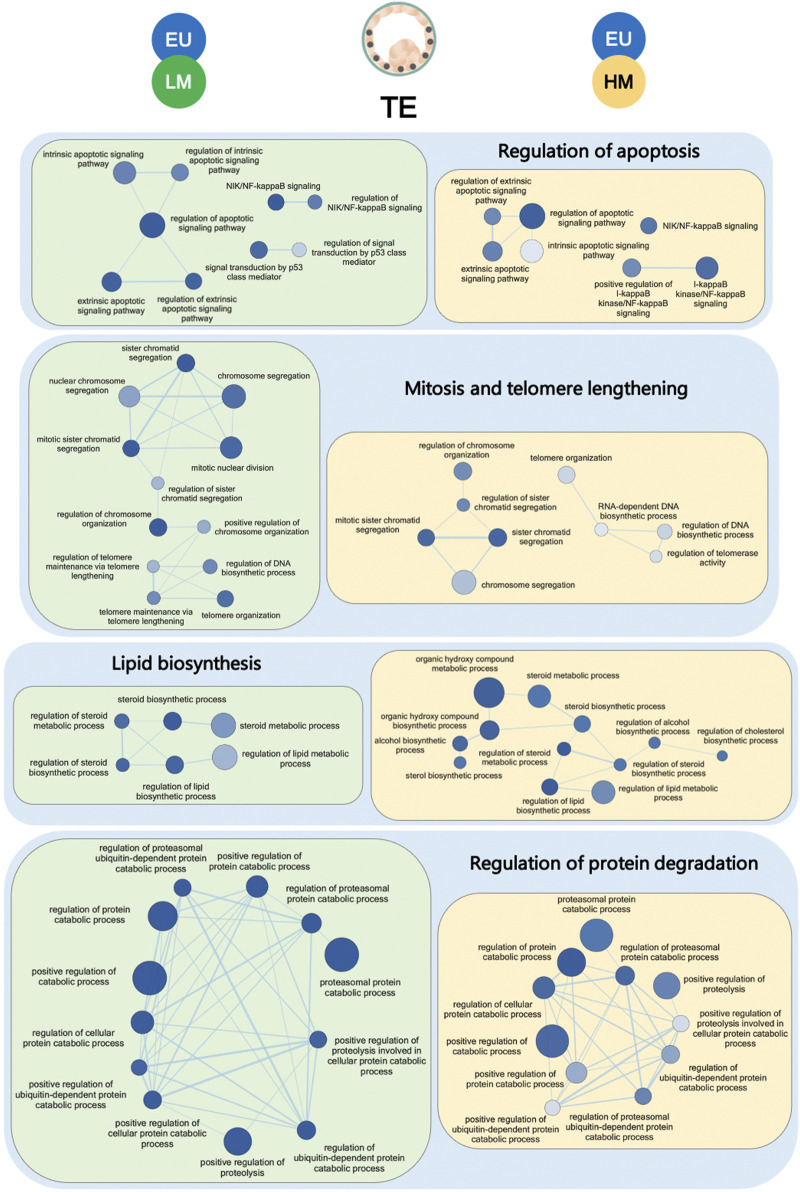
Gene set enrichment analysis of trophectoderm samples. Enrichment map of gene sets significantly downregulated in the trophectoderm (TE) of low-level mosaic embryos (LM) (green panels) and high-level mosaic embryos (HM) (yellow panels) compared to the TE of euploid (EU) embryos. Nodes represent gene ontology biological processes. Node color intensity correlates with the FDR q-value, the more intense the greater statistical significance of the difference of expression. Node size represents the number of genes in the gene set. Edges represent overlap between gene sets, and edge width represents the number of genes that overlap.

Indeed, the enrichment map of terms significantly deregulated in the TE samples of mosaic embryos revealed a global disruption of pathways involved in the regulation of apoptosis, mitosis, telomere maintenance, biosynthesis of lipids and degradation of proteins, when compared to euploid controls (Figure 3). Other disrupted pathways included those involved in WNT intercellular signaling and in the generation of precursor metabolites and energy through the TCA cycle.

#### 3.3.2 Inner cell mass cells

**FIGURE 4:**
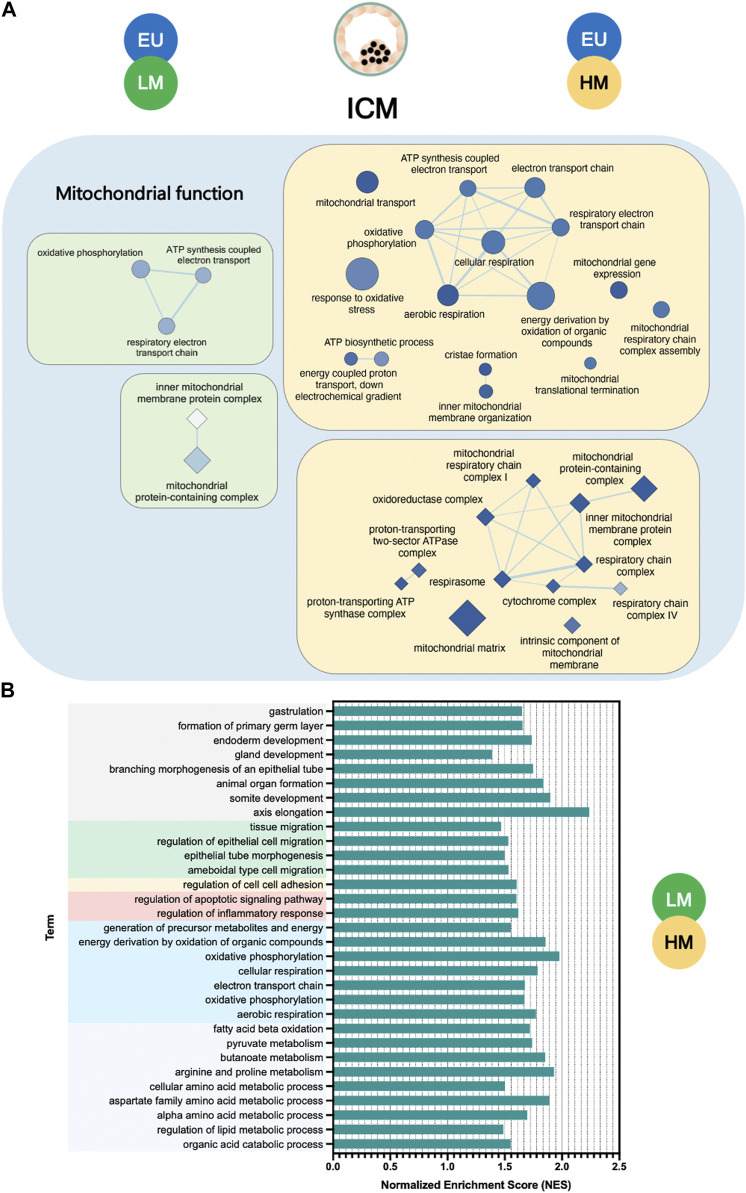
Gene set enrichment analysis of inner cell mass samples. **(A)** Enrichment map of mitochondrial terms significantly downregulated in the inner cell mass (ICM) of low-level mosaic embryos (LM) (green panels) and high-level mosaic embryos (HM) (yellow panels) compared to the ICM of euploid (EU) embryos. Nodes represent gene ontology (GO) biological processes (ellipse shape) and cellular components (diamond shape). Node color intensity correlates with the FDR q-value, the more intense the greater statistical significance of the difference of expression. Node size represents the number of genes in the gene set. Edges represent overlap between gene sets, and edge width represents the number of genes that overlap. **(B)** Biological processes and pathways significantly upregulated in the ICM of LM compared to the ICM of HM. KEGG: Kyoto Encyclopedia of Genes and Genomes.

Unlike the response observed in TE cells, we found only 12 common terms, mainly involved in mitochondrial function, to be significantly deregulated in the ICM of low-level and high-level embryos, compared to euploid controls. The extent of disruption of mitochondrial pathways, as measured by the number and statistical significance of downregulated terms, was greater in high-level mosaic embryos than in low-level mosaic embryos when compared to euploid controls (Figure 4A).

In fact, gene set enrichment analysis revealed key significant transcriptome differences (134 terms) between the ICM of low-level and high-level mosaic embryos **(** Supplementary Table S7 **).** Out of the 134 terms, 130 (97%) were upregulated in low-level mosaic embryos and were related to energy production, cellular respiration, embryonic development, cell migration and adhesion, metabolism of organic compounds, regulation of apoptosis, and inflammatory response (Figure 4B).

### 3.4 Validation of RNA-seq results by quantitative PCR

qRT-PCR was performed on a selection of DEGs (*ARHGAP36, NELL1, NTRK3, VIM*, *CDH5*, and *LAMC3*) to validate the findings of the RNA-seq experiment (Supplementary Figure S4). The results of the qRT-PCR analysis were consistent with those of RNA-seq, demonstrating significant differences in the expression of two of the six genes evaluated (*ARHGAP36* and *NELL1*), and similar trends in the remaining four genes (*NTRK3*, *VIM*, *CDH5*, and *LAMC3*). The lack of statistical significance in these genes may be due to natural variations in gene expression between individual samples.

### 3.5 Analysis of blastomere cell cycles by time lapse imaging

**FIGURE 5:**
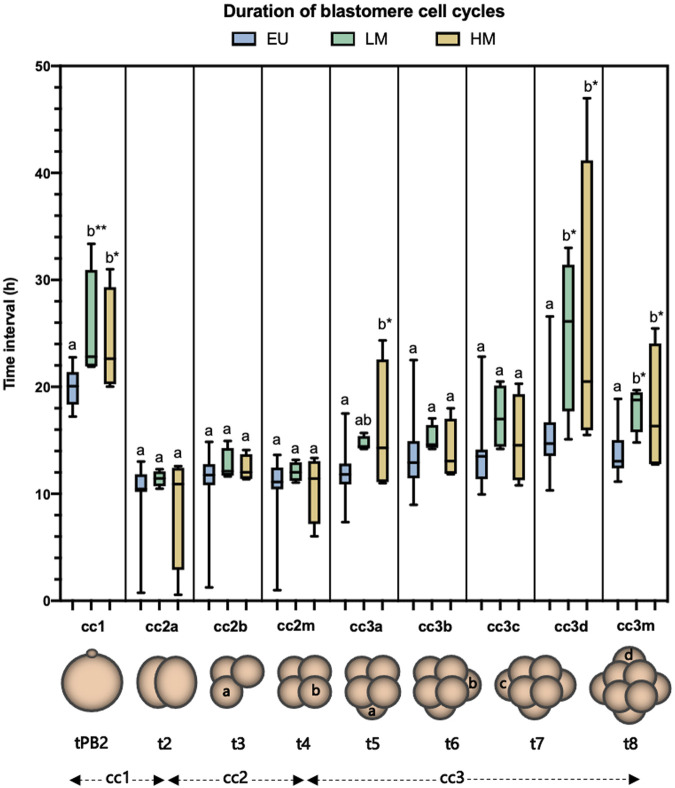
Analysis of the duration of blastomere cell cycles. Blastomere cleavage was tracked in euploid embryos (EU) (*n* = 25), low-level mosaic embryos (LM) (*n* = 4) and high-level mosaic embryos (HM) (*n* = 4). Box plot shows median, first and third quartiles, minimum, maximum and mean values. The same letters for each parameter indicate homogeneous subsets. *p ≤ 0.05; **p ≤ 0.01.

The duration of cc1 and cc3m was significantly longer in both low-level mosaic embryos (*n* = 4) (cc1 = 25.23 ± 5.49 h, *p* = 0.004; cc3m = 18.02 ± 2.19 h, *p* = 0.02) and high-level mosaic embryos (*n* = 4) (cc1 = 24.07 ± 4.97 h, *p* = 0.027; cc3m = 17.72 ± 6.13 h, *p* = 0.03), compared to euploid embryos (*n* = 25) (cc1 = 19.89 ± 1.68 h; cc3m = 13.68 ± 2.05 h). No significant morphokinetic differences were found between low-level mosaic embryos and high-level mosaic embryos (Figure 5).

## 4 Discussion

Most RNA-seq studies of the human preimplantation transcriptome have focused on investigating the transcriptional networks that govern the delineation of early cell lineages (Xue et al., 2013; Yan et al., 2013; Blakeley et al., 2015; Petropoulos et al., 2016; Boroviak et al., 2018; Stirparo et al., 2018; Meistermann et al., 2021). Other studies have attempted to identify molecular markers of embryonic competence by correlating transcriptome profiles with clinical variables related to human embryonic development, such as ploidy status or live birth potential (Kirkegaard et al., 2015; Licciardi et al., 2018; Groff et al., 2019; Ntostis et al., 2019; Sanchez-Ribas et al., 2019; Starostik et al., 2020). Furthermore, some groups have developed protocols for digital karyotyping based on gene expression data (Macaulay et al., 2015; Weissbein et al., 2016; Fan et al., 2018), some of which have been optimized for the detection of mosaic abnormalities (Griffiths et al., 2017; Starostik et al., 2020). These approaches are fundamental to understanding the molecular mechanisms of embryonic development and implantation capacity.

Transcriptome analysis of human preimplantation embryos has revealed a link between aneuploidy and different manifestations of cellular stress. Stress responses involving the deregulation of pathways related to cell proliferation, DNA damage, apoptosis, protein degradation, and mitochondrial energy metabolism have been reported in different types of samples, including whole embryos, TE biopsies, and even single cells (Regin et al., 2022). These findings underscore the complex interplay between aneuploidy and cellular stress, and suggest that the molecular mechanisms underlying this relationship are multifaceted. However, while the stress caused by the presence of aneuploid cells may compromise early development, it does not necessarily induce embryonic arrest, as both meiotic and mitotic errors can persist to the blastocyst stage (McCoy et al., 2015).

To the best of our knowledge, only one previous study has assessed the transcriptome profile of blastocysts diagnosed as mosaic after NGS-based PGT-A (Maxwell et al., 2022). In such study, global gene expression was progressively disrupted from euploid embryos (used as a control for comparisons) to mosaic embryos (with chromosomal copy numbers ranging from 20% to 80% in the TE biopsy) and aneuploid embryos (which shared the same aneuploid chromosomes as mosaic embryos).

Our aim was to investigate the impact of different levels of putative mosaicism on the gene expression profile of human blastocysts. In contrast to the previous study, all embryos were subjected to the same NGS protocol and were separated into ICM and TE fractions before RNA-seq analysis. We believe this approach to be more informative considering the critically different roles played by the ICM and the TE in promoting embryogenesis and the initiation and maintenance of implantation, respectively. Additionally, in line with recent observations regarding the reproductive capabilities of mosaic embryos (Capalbo et al., 2022), we used a threshold of 50% to differentiate between low-level and high-level mosaic embryo populations.

One important difference between our study and the previous work (Maxwell et al., 2022) is that we were unable to obtain low-level and high-level mosaic embryos with the same aneuploidies. This can be attributed to the low incidence of mosaicism at the blastocyst stage and the uncertainty surrounding the management of mosaic embryos, which led us to contact only those patients with supernumerary embryos and favorable clinical outcomes to participate in our research. Although three out of the four aneuploidies of high-level mosaic embryos were also present in low-level mosaic embryos, our data may be biased by chromosome-specific gene expression. To minimize this confounding effect, we performed gene set enrichment maps and identified global transcriptome responses to multiple aneuploidies, rather than analyzing the effects of each aneuploidy separately.

In our cohort, we found no significant association between PCAs and PGT-A categories. However, cluster heatmaps revealed a clear separation of samples according to the original diagnosis in all comparisons. The day of embryo biopsy, as well as the morphological grade of embryos, were similarly distributed across the experimental groups. This suggests that, despite the potential effect of natural variations in gene expression of individual samples or factors with undefined roles in embryo development, the original mosaic diagnoses were predictive of the remaining embryonic fractions.

These findings are supported by the identification of morphokinetic delays in several cell cycle timings within the same mosaic embryos analyzed by RNA-seq. It has been demonstrated that mitotic errors during early cell divisions can result in slower cleavage and longer cell cycles (Chavez et al., 2012), potentially due to the spindle assembly checkpoint stalling mitosis caused by incorrect chromatid alignments (Regin et al., 2022). Interestingly, the duration of cell cycles in embryos with both low-level and high-level mosaic results have been studied in detail, and timings have been found to overlap with those of euploid and aneuploid embryos (Martin et al., 2021). However, a more effective strategy for identifying cell cycle delays by TLI and providing clues about the presence of mosaicism is to track blastomere divisions individually (Grau et al., 2015).

Compared to euploid embryos, which were used as control, the gene expression distance based on the number of DEGs increased with level of mosaicism. This increase was statistically significant only in ICM samples, whereas the TE samples from low-level and high-level mosaic embryos displayed virtually equivalent gene expression profiles. Several genes related to embryo development were commonly found to be significantly deregulated in mosaic embryos, compared to euploid controls. These included ICM-related genes, such as *TRIM36* (which has been linked to chromosomal instability when overexpressed) (Miyajima et al., 2009) and *SPATA20* (which is impaired in infertile patients) (Omolaoye et al., 2022); as well as TE-related genes such as *NELL1* (which plays a role in protein modification and cellular metabolism in human preimplantation embryos) (Sun et al., 2022).

Accordingly, gene set enrichment analysis revealed a common set of pathways that were significantly deregulated in mosaic embryos compared to euploid controls. These pathways included those deregulated in TE cells such as apoptosis, cell proliferation, metabolism and protein degradation, as well as pathways deregulated in ICM cells, which were primarily related to mitochondrial function. These pathways were similar to those found in the previous study (Maxwell et al., 2022). Additionally, our findings align with previous observations demonstrating that, although the impact of mosaicism on gene expression is largely dependent on the affected chromosomes (Licciardi et al., 2018; Ntostis et al., 2019; Sanchez-Ribas et al., 2019), some reproducible effects may exist regardless of the specific chromosomal aberration (Sheltzer, 2013; Zhu et al., 2018).

The deregulation of pathways involved in apoptosis and cell proliferation observed in our study supports previous immunofluorescence studies performed in the mouse, which indicate that mosaic embryos undergo apoptosis and anti-proliferative mechanisms during the blastocyst stage (Bolton et al., 2016; Singla et al., 2020). Interestingly, in human embryos, these mechanisms appear to occur more frequently in the TE than in the ICM, as opposed to mouse embryos (Victor et al., 2019), which is also consistent with our findings. It is indeed possible that the incidence of apoptosis in human TE cells has been underestimated by the use of a single pluripotency marker (Victor et al., 2019), since such cells can retain the expression of pluripotency genes even after blastocyst expansion (Gerri et al., 2020). Furthermore, the deregulation of *p53* may serve as a specific indicator of chromosome segregation errors in apoptotic pathways (Tang, Y. et al., 2011).

Moreover, the perturbation of metabolic processes observed in the TE of mosaic embryos may be attributed to the altered stoichiometry of enzymes and their regulators caused by aneuploidy (Zhu et al., 2018; Chunduri and Storchová, 2019; Groff et al., 2019). The disruption of pathways involved in protein degradation by the ubiquitin-proteasome system in the TE of mosaic blastocysts suggests proteotoxic stress resulting from the accumulation of unfolded proteins, which is a well-known consequence of transcriptome deregulation (Walter and Ron, 2011). Additionally, deregulation of telomere elongation pathways in the TE might reflect potential differences in telomere maintenance dynamics across cell lineages, as reported in other studies (Iqbal et al., 2011; Varela et al., 2011; Kalmbach et al., 2014).

At the ICM level, pathways related to mitochondria were found to be commonly deregulated in both low-level and high-level mosaic embryos, when compared to euploid controls. The presence of aneuploid cells in the embryo could lead to mitochondrial dysfunction through various mechanisms. For instance, trisomy 21 can alter the expression of genes involved in mitochondrial biogenesis and function, which in turn can result in mitochondrial defects (Izzo et al., 2018). Additionally, chromosomal alterations can lead to the accumulation of reactive oxygen species, which can disrupt mitochondrial membrane potential, reduce ATP production, and impair the electron transport chain, ultimately leading to cellular stress (Zhu et al., 2018).

Unlike what was observed in TE cells, the gene expression profiles of low-level mosaic embryos and high-level mosaic embryos differed significantly at the ICM level. This difference in gene expression involved the significant upregulation of the genes *ALDH2, GPX2*, and *CDH5* in low-level mosaic embryos. These genes are key for embryo implantation and pregnancy (Zhou et al., 1997; Roland-Zejly et al., 2011; Sata et al., 2012; Kinnear et al., 2019). Indeed, they belong to the “embryo implantation universe” proposed by (Sanchez-Ribas et al., 2019). Intriguingly, these genes were also significantly upregulated in euploid embryos compared to high-level mosaic embryos, but their expression levels were comparable between euploid embryos and low-level mosaic embryos. A similar expression pattern was observed in the hypoblast marker *SOX17*, supporting the notion that aneuploidy can alter cell fate decisions during early embryonic development and lead to defects in cell-lineage formation (Shahbazi et al., 2020).

Consistently, pathways involving the formation of the primary germ layer, endoderm development, energy production, metabolism of organic compounds, and regulation of apoptosis were significantly upregulated in the ICM of low-level mosaic embryos compared to high-level mosaic embryos.

Taken together, these findings challenge the notion that embryos with mosaic results are simply a by-product of misdiagnosis due to false positive mosaic calls. While this might be the most common scenario, the reproductive potential of these embryos may also be explained by the complex interplay of molecular mechanisms which become deregulated in the presence of aneuploid cells. Our findings also suggest that within mosaic embryos, those with low-level mosaicism may exhibit transcriptome profiles more closely resembling those of euploid embryos, while high-level mosaic embryos represent a distinct biological category characterized by molecular features indicative of a greater extent and burden of the cellular stress arising from aneuploidy. These findings align well with current perspectives on the reproductive potential of mosaic embryos, and contribute to the growing body of evidence suggesting that low and high levels of mosaicism after PGT-A deserve distinct clinical management.

Some limitations of our study should be acknowledged. First, given the difficulty of obtaining embryos with the highest morphological quality for research purposes, our analysis was restricted to embryos graded B or C. In addition, global gene expression may exhibit high heterogeneity even among high-quality embryos at the same developmental stage (Shaw et al., 2013; Ntostis et al., 2019). This means that our results may not be generalizable to other embryo populations, especially those with higher morphological scores. Furthermore, beyond the potential effect of chromosome-specific gene expression, the impact of mosaic aneuploidies on global transcriptome profiles may be confounded by specific adaptive mechanisms, particularly considering the extraordinary plasticity of early human development (Coticchio et al., 2021). Finally, while transcriptomics can serve as a sensitive indicator of cellular stress, it provides little to no information on protein activity. Therefore, our results should be interpreted with caution, and further studies focusing on protein are necessary to confirm these findings.

In conclusion, our study demonstrates that mosaic results following PGT-A may be accompanied by significant alterations in gene expression across blastocyst compartments as well as by significant delays in cell cycle timings during the first mitotic divisions. The level of mosaicism was associated with the extent of transcriptome deregulation, and pathways involving apoptosis, mitosis, protein degradation, metabolism, and mitochondrial energy production were among the most deregulated within mosaic embryos. We believe that these findings contribute towards the molecular characterization of mosaic embryos and offer new insights into the factors that determine their developmental potential.

## Data Availability

The datasets presented in this study can be found in online repositories. The name of the repository and accession number can be found below: Gene Expression Omnibus (GEO); GSE194306 [https://www.ncbi.nlm.nih.gov/geo/query/acc.cgi?acc=GSE194306].
