# Supplementary material for: Mosaic results after preimplantation genetic testing for aneuploidy may be accompanied by changes in global gene expression
Source: Front Mol Biosci. 2023 Apr 14;10:1180689. doi: 10.3389/fmolb.2023.1180689 (PMC10140421; doi:10.3389/fmolb.2023.1180689)
Supplement: Supplementary file 1 [file DataSheet1.DOCX]

Supplementary Material

Mosaic results after preimplantation genetic testing for aneuploidy may be accompanied by changes in global gene expression

**A. Martin, A. Mercader, F. Dominguez, A. Quiñonero, M. Perez, R. Gonzalez-Martin, A. Delgado, A. Mifsud, A. Pellicer, M.J. De los Santos^*^**

*** Correspondence:** María José de los Santos: MariaJose.DelosSantos@ivirma.com

# Supplementary Figures and Tables

## Supplementary Figures


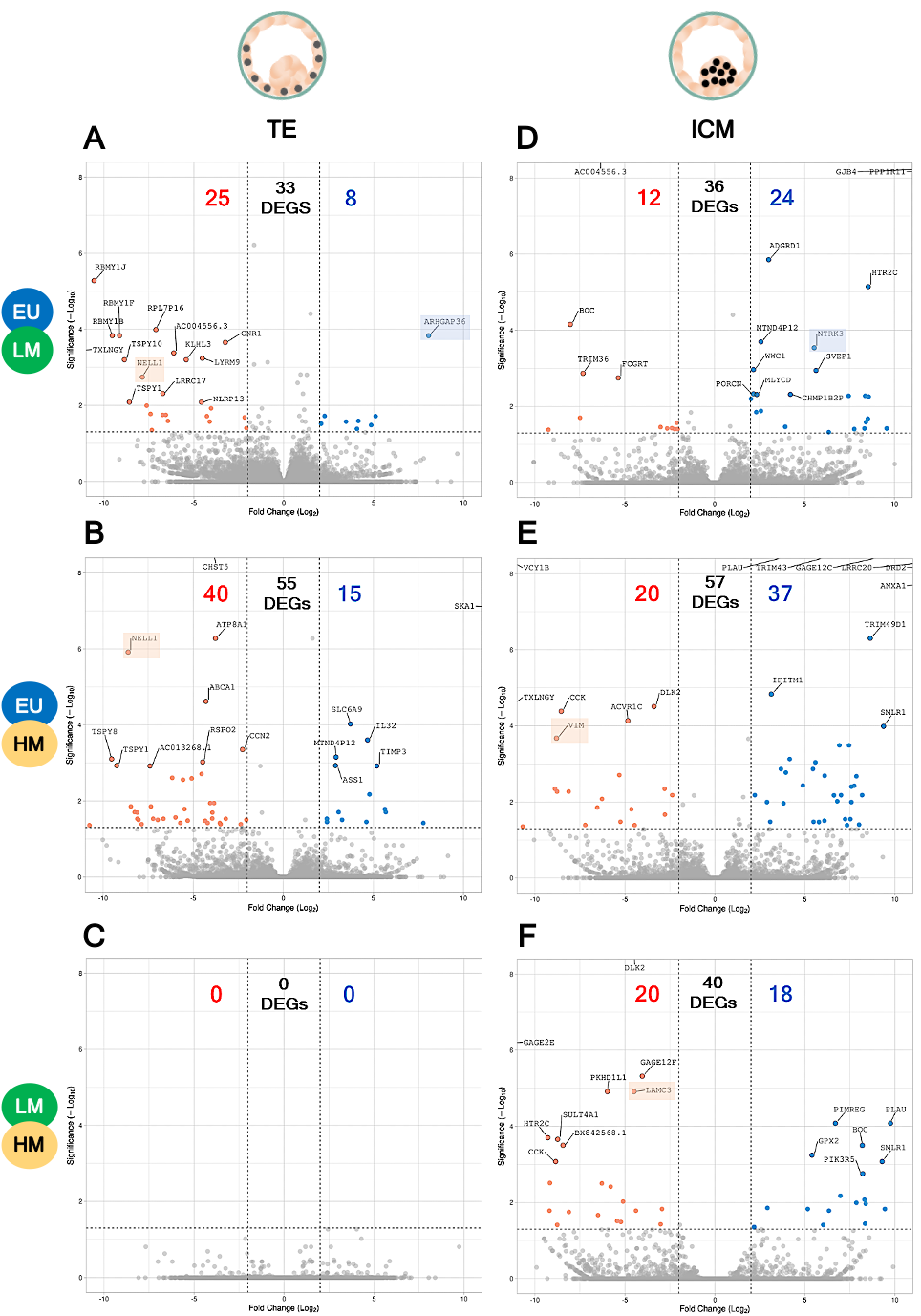


**Supplementary Figure 1.** **Volcano plot of significantly differentially expressed genes (DEGs).** **(A-C)** Trophectoderm comparisons (TE). **(D-F)** Inner cell mass comparisons (ICM). The number of DEGs are indicated for each comparison. Red: DEGs downregulated in the first comparison factor. Blue: DEGs upregulated in the first comparison factor. DEGs selected for validation by quantitative PCR are highlighted. EU: euploid. LM: low-level mosaic. HM: high-level mosaic.


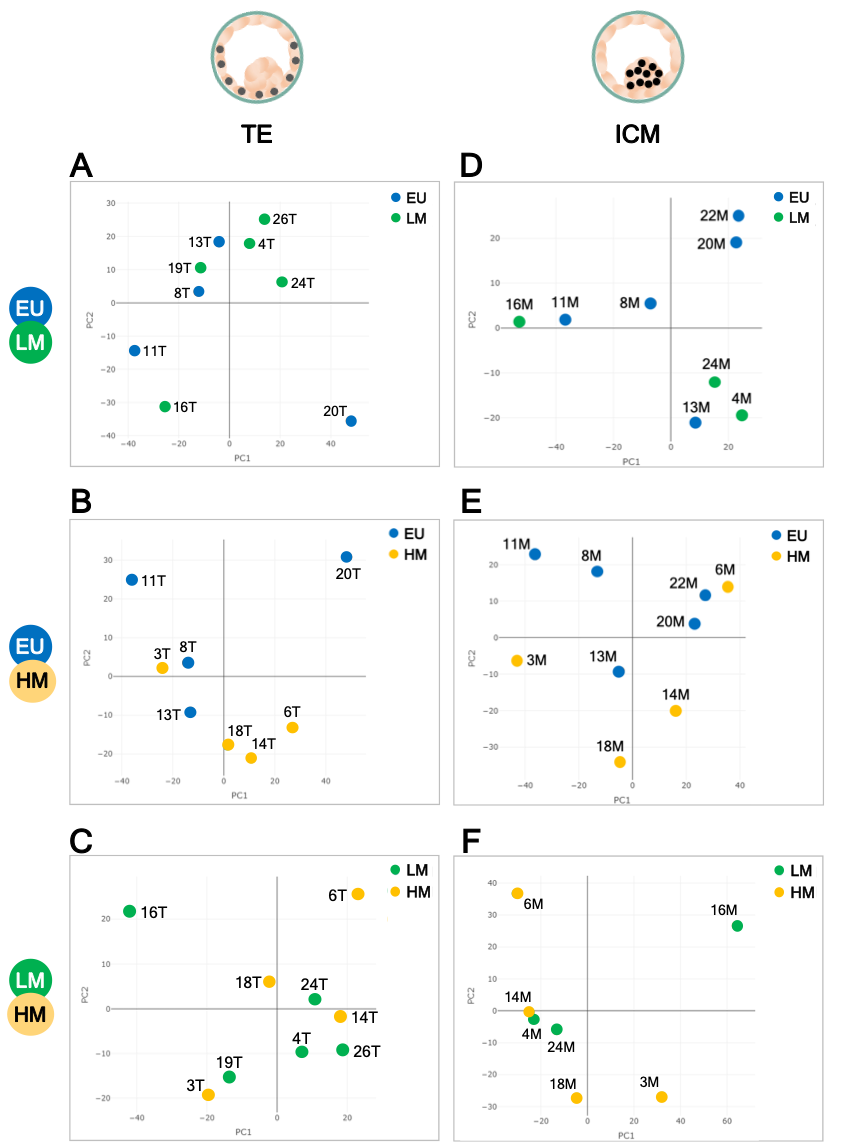


**Supplementary Figure 2.** **Principal Component Analysis.** **(A-C)** Trophectoderm comparisons (TE). **(D-F)** Inner cell mass comparisons (ICM). EU: euploid. LM: low-level mosaic. HM: high-level mosaic.


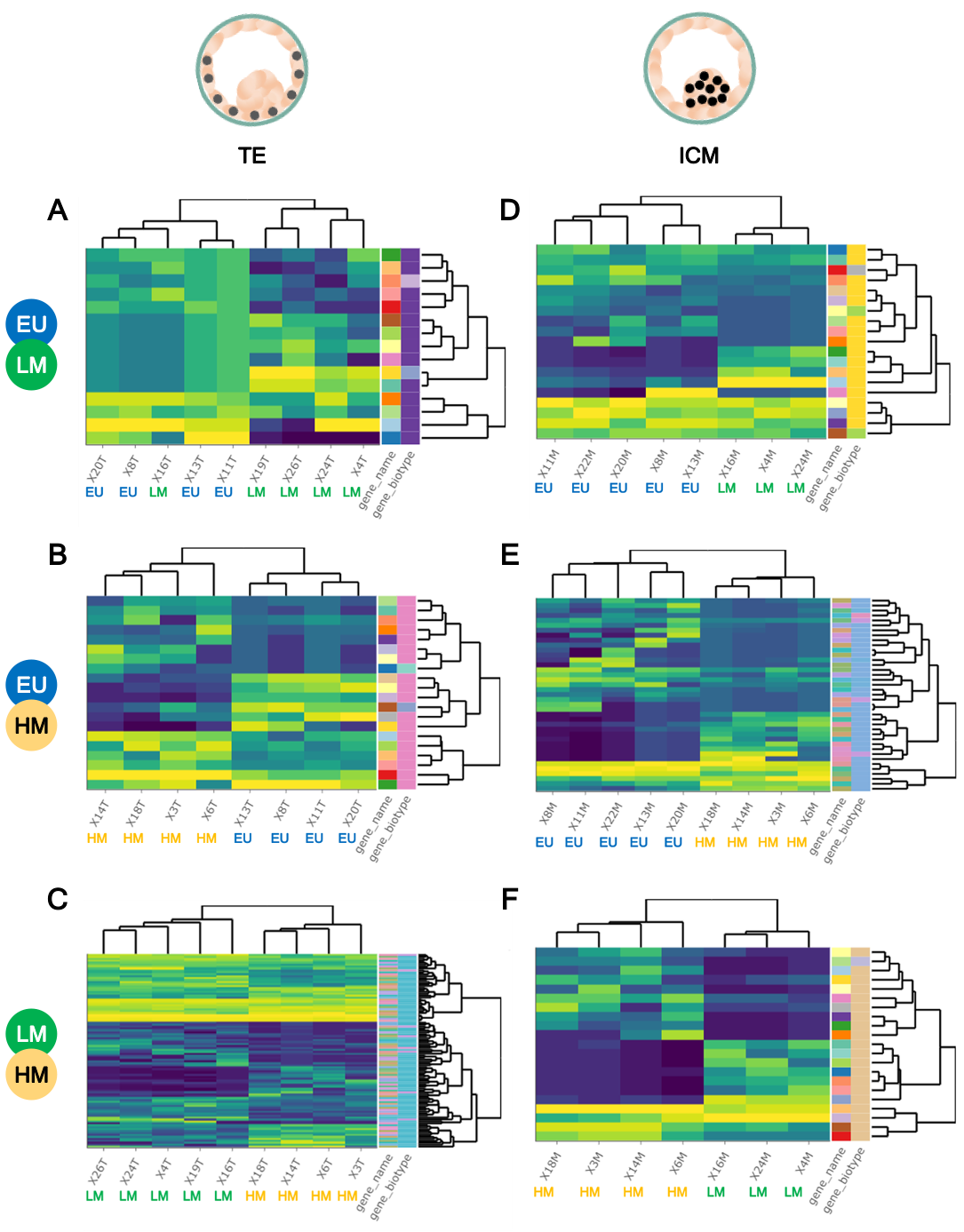


**Supplementary Figure 3.** **Heatmap and hierarchical clustering.** **(A-C)** Trophectoderm comparisons (TE). **(D-F)** Inner cell mass comparisons (ICM). Normalized counts were obtained by dividing the raw counts of each gene by the size factor, which was calculated by the median ratio method. Yellow lines represent higher abundance, and blue lines represent lower abundance compared with the other condition. EU: euploid. LM: low-level mosaic. HM: high-level mosaic.


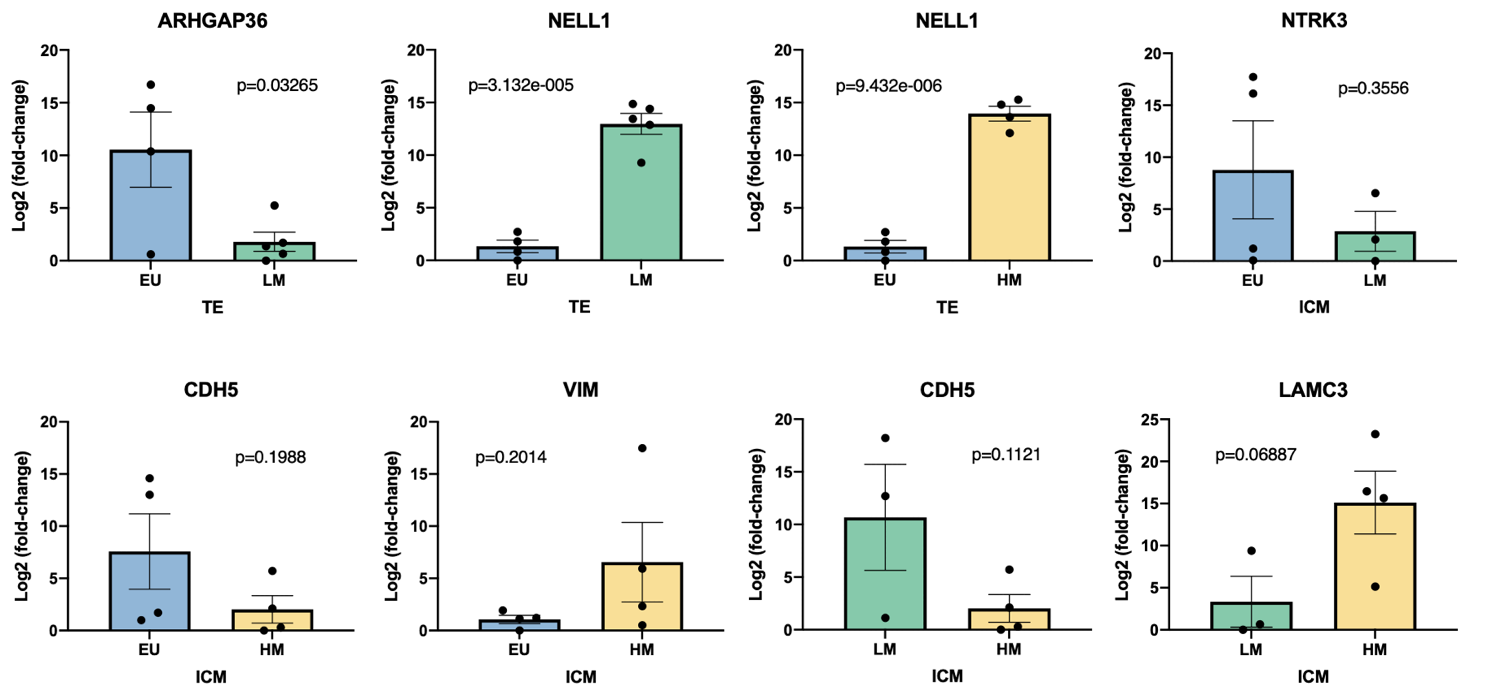


**Supplementary Figure 4: Validation of RNA-seq by quantitative PCR.** The expression levels of the selected genes ARHGAP36, NELL1, NTRK3, CDH5, VIM and LAMC3 were validated by quantitative PCR, quantified by the ΔΔCT method and expressed as fold regulation. Statistical analysis was performed by using a t-test for independent samples (two-tailed). *p<0.05; **p<0.01; ***p<0.001 were considered statistically significant. ICM: inner cell mass; TE: trophectoderm; EU: euploid; LM: low-level mosaic; HM: high-level mosaic.

## Supplementary Tables

**Supplementary Table 1. List of primer sequences used for the validation of the RNA-seq results by quantitative PCR.** FW, forward primer; RV, reverse primer.

| **Name** | **Primer sequence ( 5'->3' )** |
| --- | --- |
| NTRK3 FW | GTGAGTCCCACACCTCCTATC |
| NTRK3 RV | AAGCAGCAAGTCCAACTGCTA |
| CDH5 FW | CCAGCCCAAAGTGTGTGAGA |
| CDH5 RV | TGGTATGCTCCCGGTCAAAC |
| VIM FW | GTGGACCAGCTAACCAACGA |
| VIM RV | GCCAGAGACGCATTGTCAAC |
| LAMC3 FW | AGCAATGTCCGTCCTGCTAC |
| LAMC3 RV | CCCCTCCGTCAAAGTCAGTC |
| ARHGAP36 FW | ACTTGGCCTTGGTGTTTGGA |
| ARHGAP36 RV | ATCATGGCACGGACCACATT |
| NELL1 FW | CTTTGGGATGGACCCTGACC |
| NELL1 RV | TCACATGAGGAGCTGCATGG |

**Supplementary Table 2. List of all differentially expressed genes found across the six comparisons analyzed.** Genes with a Benjamini-Hochberg adjusted p-value (padj) < 0.05 and a log2foldchange of ±2 was considered significantly differentially expressed. log2FoldChange > 0 indicates significant upregulation in the first comparison factor. EU: euploid. LM: low-level mosaic. HM: high-level mosaic. TE: trophectoderm. ICM: inner cell mass.

| **Fraction** | **Comparison** | **Gene symbol** | **Gene biotype** | **log2FoldChange** | **padj** |
| --- | --- | --- | --- | --- | --- |
| TE | (C-1) EU-LM | ARHGAP36 | protein coding | 8,06 | 1,5E-04 |
| TE | (C-1) EU-LM | KLF4P1 | pseudogene | 2,27 | 1,9E-02 |
| TE | (C-1) EU-LM | PAGE5 | protein coding | 5,10 | 1,9E-02 |
| TE | (C-1) EU-LM | MTND6P4 | pseudogene | 4,15 | 2,6E-02 |
| TE | (C-1) EU-LM | RPP21 | protein coding | 3,47 | 2,7E-02 |
| TE | (C-1) EU-LM | GUSBP4 | pseudogene | 2,08 | 3,0E-02 |
| TE | (C-1) EU-LM | DGKK | protein coding | 4,86 | 3,3E-02 |
| TE | (C-1) EU-LM | CXorf49 | protein coding | 4,08 | 4,1E-02 |
| TE | (C-1) EU-LM | RBMY1J | protein coding | -10,55 | 5,3E-06 |
| TE | (C-1) EU-LM | RPL7P16 | pseudogene | -7,11 | 1,0E-04 |
| TE | (C-1) EU-LM | RBMY1F | protein coding | -9,13 | 1,5E-04 |
| TE | (C-1) EU-LM | RBMY1B | protein coding | -9,54 | 1,5E-04 |
| TE | (C-1) EU-LM | CNR1 | protein coding | -3,25 | 2,2E-04 |
| TE | (C-1) EU-LM | TXLNGY | pseudogene | -11,76 | 3,6E-04 |
| TE | (C-1) EU-LM | AC004556.3 | protein coding | -6,12 | 4,2E-04 |
| TE | (C-1) EU-LM | LYRM9 | protein coding | -4,53 | 5,8E-04 |
| TE | (C-1) EU-LM | KLHL3 | protein coding | -5,42 | 6,3E-04 |
| TE | (C-1) EU-LM | TSPY10 | protein coding | -8,86 | 6,3E-04 |
| TE | (C-1) EU-LM | NELL1 | protein coding | -7,86 | 1,8E-03 |
| TE | (C-1) EU-LM | LRRC17 | protein coding | -6,73 | 4,8E-03 |
| TE | (C-1) EU-LM | NLRP13 | protein coding | -4,58 | 8,2E-03 |
| TE | (C-1) EU-LM | TSPY1 | protein coding | -8,59 | 8,2E-03 |
| TE | (C-1) EU-LM | RBMY2FP | pseudogene | -7,62 | 1,0E-02 |
| TE | (C-1) EU-LM | PKDCC | protein coding | -4,04 | 1,2E-02 |
| TE | (C-1) EU-LM | HAL | protein coding | -7,41 | 1,7E-02 |
| TE | (C-1) EU-LM | COL24A1 | protein coding | -6,56 | 1,8E-02 |
| TE | (C-1) EU-LM | RBMY1E | protein coding | -6,73 | 1,8E-02 |
| TE | (C-1) EU-LM | CPNE4 | protein coding | -4,27 | 1,9E-02 |
| TE | (C-1) EU-LM | KBTBD3 | protein coding | -2,18 | 2,1E-02 |
| TE | (C-1) EU-LM | MAGEA8 | protein coding | -6,44 | 2,6E-02 |
| TE | (C-1) EU-LM | THBS4 | protein coding | -4,12 | 2,7E-02 |
| TE | (C-1) EU-LM | IGKC | protein coding | -2,08 | 4,0E-02 |
| TE | (C-1) EU-LM | NCR1 | protein coding | -7,34 | 4,5E-02 |
| TE | (C-2) EU-HM | SKA1 | protein coding | 22,93 | 1,2E-07 |
| TE | (C-2) EU-HM | SLC6A9 | protein coding | 3,73 | 9,4E-05 |
| TE | (C-2) EU-HM | IL32 | protein coding | 4,68 | 2,5E-04 |
| TE | (C-2) EU-HM | MTND4P12 | pseudogene | 2,93 | 7,0E-04 |
| TE | (C-2) EU-HM | ASS1 | protein coding | 2,90 | 1,2E-03 |
| TE | (C-2) EU-HM | TIMP3 | protein coding | 5,19 | 1,2E-03 |
| TE | (C-2) EU-HM | DGKK | protein coding | 4,79 | 6,7E-03 |
| TE | (C-2) EU-HM | TRPV2 | protein coding | 5,64 | 1,6E-02 |
| TE | (C-2) EU-HM | TMEM176B | protein coding | 5,69 | 2,0E-02 |
| TE | (C-2) EU-HM | RASGRP1 | protein coding | 3,07 | 2,0E-02 |
| TE | (C-2) EU-HM | C4orf33 | protein coding | 2,42 | 2,9E-02 |
| TE | (C-2) EU-HM | ITPRIP | protein coding | 3,26 | 3,1E-02 |
| TE | (C-2) EU-HM | CYP19A1 | protein coding | 4,61 | 3,5E-02 |
| TE | (C-2) EU-HM | POGLUT1 | protein coding | 2,42 | 3,5E-02 |
| TE | (C-2) EU-HM | LOXL4 | protein coding | 7,78 | 3,8E-02 |
| TE | (C-2) EU-HM | CHST5 | protein coding | -3,95 | 1,7E-09 |
| TE | (C-2) EU-HM | ATP8A1 | protein coding | -3,77 | 5,3E-07 |
| TE | (C-2) EU-HM | NELL1 | protein coding | -8,61 | 1,2E-06 |
| TE | (C-2) EU-HM | ABCA1 | protein coding | -4,29 | 2,4E-05 |
| TE | (C-2) EU-HM | CCN2 | protein coding | -2,26 | 4,5E-04 |
| TE | (C-2) EU-HM | TSPY8 | protein coding | -9,53 | 7,9E-04 |
| TE | (C-2) EU-HM | RSPO2 | protein coding | -4,47 | 9,4E-04 |
| TE | (C-2) EU-HM | TSPY1 | protein coding | -9,24 | 1,2E-03 |
| TE | (C-2) EU-HM | AC013268.1 | pseudogene | -7,40 | 1,2E-03 |
| TE | (C-2) EU-HM | PKHD1L1 | protein coding | -4,53 | 1,9E-03 |
| TE | (C-2) EU-HM | GABRA3 | protein coding | -6,16 | 2,5E-03 |
| TE | (C-2) EU-HM | ADCY5 | protein coding | -5,08 | 2,5E-03 |
| TE | (C-2) EU-HM | CPNE4 | protein coding | -5,57 | 2,8E-03 |
| TE | (C-2) EU-HM | ZP4 | protein coding | -3,85 | 1,1E-02 |
| TE | (C-2) EU-HM | GYG2P1 | pseudogene | -4,05 | 1,1E-02 |
| TE | (C-2) EU-HM | TSPY9P | protein coding | -8,46 | 1,4E-02 |
| TE | (C-2) EU-HM | RPL7P16 | pseudogene | -7,35 | 1,4E-02 |
| TE | (C-2) EU-HM | TEX101 | protein coding | -5,48 | 1,6E-02 |
| TE | (C-2) EU-HM | MAGEA8 | protein coding | -8,25 | 2,0E-02 |
| TE | (C-2) EU-HM | TXLNGY | pseudogene | -12,20 | 2,0E-02 |
| TE | (C-2) EU-HM | SLC30A8 | protein coding | -3,93 | 2,0E-02 |
| TE | (C-2) EU-HM | AC008758.1 | protein coding | -8,11 | 2,0E-02 |
| TE | (C-2) EU-HM | GRIN2B | protein coding | -5,98 | 2,7E-02 |
| TE | (C-2) EU-HM | LEF1 | protein coding | -3,94 | 2,9E-02 |
| TE | (C-2) EU-HM | CD37 | protein coding | -3,19 | 2,9E-02 |
| TE | (C-2) EU-HM | HBZ | protein coding | -7,22 | 2,9E-02 |
| TE | (C-2) EU-HM | SHISAL2B | protein coding | -6,65 | 2,9E-02 |
| TE | (C-2) EU-HM | TSPY3 | protein coding | -8,10 | 2,9E-02 |
| TE | (C-2) EU-HM | MARCHF2 | protein coding | -2,05 | 3,1E-02 |
| TE | (C-2) EU-HM | TNFRSF10C | protein coding | -6,97 | 3,1E-02 |
| TE | (C-2) EU-HM | GAGE2E | protein coding | -8,04 | 3,1E-02 |
| TE | (C-2) EU-HM | EBF1 | protein coding | -5,30 | 3,3E-02 |
| TE | (C-2) EU-HM | PIMREG | protein coding | -4,33 | 3,3E-02 |
| TE | (C-2) EU-HM | PHACTR3 | protein coding | -3,52 | 3,8E-02 |
| TE | (C-2) EU-HM | SRRM3 | protein coding | -5,72 | 3,8E-02 |
| TE | (C-2) EU-HM | NLRP13 | protein coding | -4,20 | 3,8E-02 |
| TE | (C-2) EU-HM | PDGFRL | protein coding | -3,48 | 4,1E-02 |
| TE | (C-2) EU-HM | RBMY2FP | pseudogene | -7,86 | 4,1E-02 |
| TE | (C-2) EU-HM | TDRD6 | protein coding | -2,36 | 4,1E-02 |
| TE | (C-2) EU-HM | RBMY1J | protein coding | -10,76 | 4,3E-02 |
| ICM | (C-4) EU-LM | GJB4 | protein coding | 22,53 | 3,7E-09 |
| ICM | (C-4) EU-LM | PPP1R11 | protein coding | 25,41 | 3,7E-09 |
| ICM | (C-4) EU-LM | ADGRD1 | protein coding | 3,01 | 1,4E-06 |
| ICM | (C-4) EU-LM | HTR2C | protein coding | 8,54 | 7,1E-06 |
| ICM | (C-4) EU-LM | MTND4P12 | pseudogene | 2,58 | 2,0E-04 |
| ICM | (C-4) EU-LM | NTRK3 | protein coding | 5,54 | 2,9E-04 |
| ICM | (C-4) EU-LM | WWC1 | protein coding | 2,16 | 1,1E-03 |
| ICM | (C-4) EU-LM | SVEP1 | protein coding | 5,64 | 1,1E-03 |
| ICM | (C-4) EU-LM | PORCN | protein coding | 2,16 | 4,7E-03 |
| ICM | (C-4) EU-LM | CHMP1B2P | pseudogene | 4,21 | 4,8E-03 |
| ICM | (C-4) EU-LM | MLYCD | protein coding | 2,34 | 4,9E-03 |
| ICM | (C-4) EU-LM | C10orf90 | protein coding | 8,38 | 5,3E-03 |
| ICM | (C-4) EU-LM | MTND1P32 | pseudogene | 7,45 | 5,3E-03 |
| ICM | (C-4) EU-LM | AL590764.2 | protein coding | 8,57 | 5,5E-03 |
| ICM | (C-4) EU-LM | NOTCH2NLB | protein coding | 2,02 | 6,4E-03 |
| ICM | (C-4) EU-LM | SLC12A7 | protein coding | 2,58 | 1,3E-02 |
| ICM | (C-4) EU-LM | LRRC6 | protein coding | 2,32 | 1,4E-02 |
| ICM | (C-4) EU-LM | AC080023.2 | pseudogene | 8,52 | 2,1E-02 |
| ICM | (C-4) EU-LM | BX664615.2 | protein coding | 8,42 | 2,6E-02 |
| ICM | (C-4) EU-LM | ANK1 | protein coding | 3,93 | 3,5E-02 |
| ICM | (C-4) EU-LM | SPATA20 | protein coding | 8,33 | 3,8E-02 |
| ICM | (C-4) EU-LM | ANKK1 | protein coding | 9,57 | 3,8E-02 |
| ICM | (C-4) EU-LM | RNF14P1 | pseudogene | 7,77 | 4,0E-02 |
| ICM | (C-4) EU-LM | RPL10P9 | pseudogene | 6,35 | 4,8E-02 |
| ICM | (C-4) EU-LM | AC004556.3 | protein coding | -6,54 | 3,1E-71 |
| ICM | (C-4) EU-LM | BOC | protein coding | -8,02 | 7,0E-05 |
| ICM | (C-4) EU-LM | TRIM36 | protein coding | -7,32 | 1,4E-03 |
| ICM | (C-4) EU-LM | FCGRT | protein coding | -5,36 | 1,8E-03 |
| ICM | (C-4) EU-LM | AHNAK2 | protein coding | -7,49 | 2,0E-02 |
| ICM | (C-4) EU-LM | H2BC15 | protein coding | -2,10 | 2,7E-02 |
| ICM | (C-4) EU-LM | PCDH9 | protein coding | -3,01 | 3,5E-02 |
| ICM | (C-4) EU-LM | PIK3R2 | protein coding | -2,38 | 3,7E-02 |
| ICM | (C-4) EU-LM | TSPAN31 | protein coding | -2,64 | 3,8E-02 |
| ICM | (C-4) EU-LM | PMAIP1 | protein coding | -2,05 | 4,0E-02 |
| ICM | (C-4) EU-LM | CNNM2 | protein coding | -2,22 | 4,0E-02 |
| ICM | (C-4) EU-LM | RBMY1J | protein coding | -9,24 | 4,1E-02 |
| ICM | (C-5) EU-HM | PLAU | protein coding | 22,33 | 2,5E-11 |
| ICM | (C-5) EU-HM | TRIM43 | protein coding | 22,19 | 3,6E-11 |
| ICM | (C-5) EU-HM | GAGE12C | protein coding | 24,64 | 9,6E-11 |
| ICM | (C-5) EU-HM | LRRC20 | protein coding | 21,61 | 1,7E-09 |
| ICM | (C-5) EU-HM | DRD2 | protein coding | 21,42 | 4,7E-09 |
| ICM | (C-5) EU-HM | ANXA1 | protein coding | 22,62 | 5,8E-09 |
| ICM | (C-5) EU-HM | TRIM49D1 | protein coding | 8,63 | 5,0E-07 |
| ICM | (C-5) EU-HM | IFITM1 | protein coding | 3,13 | 1,5E-05 |
| ICM | (C-5) EU-HM | SMLR1 | protein coding | 9,37 | 1,0E-04 |
| ICM | (C-5) EU-HM | CPXM2 | protein coding | 6,91 | 3,3E-04 |
| ICM | (C-5) EU-HM | SLC28A1 | protein coding | 7,45 | 3,3E-04 |
| ICM | (C-5) EU-HM | GPX2 | protein coding | 4,15 | 7,4E-04 |
| ICM | (C-5) EU-HM | SLPI | protein coding | 5,58 | 9,1E-04 |
| ICM | (C-5) EU-HM | KLK13 | protein coding | 5,44 | 1,4E-03 |
| ICM | (C-5) EU-HM | GVINP1 | pseudogene | 3,67 | 1,4E-03 |
| ICM | (C-5) EU-HM | GLRX | protein coding | 3,94 | 1,7E-03 |
| ICM | (C-5) EU-HM | HNRNPA1P21 | pseudogene | 6,10 | 2,0E-03 |
| ICM | (C-5) EU-HM | RPL10P9 | pseudogene | 7,86 | 2,1E-03 |
| ICM | (C-5) EU-HM | MTND6P4 | pseudogene | 4,89 | 3,7E-03 |
| ICM | (C-5) EU-HM | AP000356.3 | pseudogene | 7,74 | 3,7E-03 |
| ICM | (C-5) EU-HM | MSTO2P | pseudogene | 7,59 | 4,0E-03 |
| ICM | (C-5) EU-HM | ALDH2 | protein coding | 2,22 | 6,6E-03 |
| ICM | (C-5) EU-HM | CDH5 | protein coding | 8,18 | 6,6E-03 |
| ICM | (C-5) EU-HM | C10orf90 | protein coding | 7,01 | 6,6E-03 |
| ICM | (C-5) EU-HM | SEPTIN6 | protein coding | 6,61 | 6,7E-03 |
| ICM | (C-5) EU-HM | HNRNPA1P16 | pseudogene | 6,80 | 9,6E-03 |
| ICM | (C-5) EU-HM | GPR1 | protein coding | 2,89 | 1,0E-02 |
| ICM | (C-5) EU-HM | AC093809.1 | pseudogene | 7,58 | 1,0E-02 |
| ICM | (C-5) EU-HM | SLC6A9 | protein coding | 3,81 | 1,1E-02 |
| ICM | (C-5) EU-HM | SPATA20 | protein coding | 7,24 | 2,8E-02 |
| ICM | (C-5) EU-HM | CAMKV | protein coding | 7,53 | 2,8E-02 |
| ICM | (C-5) EU-HM | SOX17 | protein coding | 6,08 | 3,1E-02 |
| ICM | (C-5) EU-HM | MT1H | protein coding | 3,07 | 3,3E-02 |
| ICM | (C-5) EU-HM | APOBEC3C | protein coding | 5,48 | 3,3E-02 |
| ICM | (C-5) EU-HM | PRDM11 | protein coding | 5,78 | 3,4E-02 |
| ICM | (C-5) EU-HM | PAPOLB | protein coding | 8,01 | 3,9E-02 |
| ICM | (C-5) EU-HM | GBA3 | pseudogene | 7,34 | 4,1E-02 |
| ICM | (C-5) EU-HM | VCY1B | protein coding | -27,01 | 4,8E-13 |
| ICM | (C-5) EU-HM | DLK2 | protein coding | -3,38 | 3,1E-05 |
| ICM | (C-5) EU-HM | CCK | protein coding | -8,54 | 4,1E-05 |
| ICM | (C-5) EU-HM | TXLNGY | pseudogene | -11,89 | 4,7E-05 |
| ICM | (C-5) EU-HM | ACVR1C | protein coding | -4,83 | 7,3E-05 |
| ICM | (C-5) EU-HM | VIM | protein coding | -8,80 | 2,1E-04 |
| ICM | (C-5) EU-HM | FCGRT | protein coding | -5,31 | 2,0E-03 |
| ICM | (C-5) EU-HM | JAG1 | protein coding | -2,79 | 4,5E-03 |
| ICM | (C-5) EU-HM | ITIH6 | protein coding | -8,88 | 4,5E-03 |
| ICM | (C-5) EU-HM | MORC1 | protein coding | -8,12 | 5,3E-03 |
| ICM | (C-5) EU-HM | TSPY1 | protein coding | -8,77 | 5,3E-03 |
| ICM | (C-5) EU-HM | CNNM2 | protein coding | -2,37 | 6,6E-03 |
| ICM | (C-5) EU-HM | ARHGEF28 | protein coding | -6,27 | 8,3E-03 |
| ICM | (C-5) EU-HM | TRIM36 | protein coding | -6,53 | 1,4E-02 |
| ICM | (C-5) EU-HM | H2BC20P | pseudogene | -4,64 | 1,5E-02 |
| ICM | (C-5) EU-HM | CXCL16 | protein coding | -2,78 | 2,1E-02 |
| ICM | (C-5) EU-HM | NELL1 | protein coding | -5,25 | 3,3E-02 |
| ICM | (C-5) EU-HM | NPHP3-ACAD11 | protein coding | -7,20 | 4,0E-02 |
| ICM | (C-5) EU-HM | PON1 | protein coding | -4,46 | 4,1E-02 |
| ICM | (C-5) EU-HM | RBMY1J | protein coding | -10,68 | 4,4E-02 |
| ICM | (C-6) LM-HM | PLAU | protein coding | 9,75 | 8,4E-05 |
| ICM | (C-6) LM-HM | PIMREG | protein coding | 6,69 | 8,4E-05 |
| ICM | (C-6) LM-HM | BOC | protein coding | 8,19 | 3,2E-04 |
| ICM | (C-6) LM-HM | GPX2 | protein coding | 5,39 | 5,7E-04 |
| ICM | (C-6) LM-HM | SMLR1 | protein coding | 9,29 | 8,5E-04 |
| ICM | (C-6) LM-HM | PIK3R5 | protein coding | 8,21 | 1,7E-03 |
| ICM | (C-6) LM-HM | LGALS2 | protein coding | 6,97 | 6,7E-03 |
| ICM | (C-6) LM-HM | CDH5 | protein coding | 8,31 | 8,5E-03 |
| ICM | (C-6) LM-HM | LMLN2 | protein coding | 7,86 | 1,0E-02 |
| ICM | (C-6) LM-HM | PKIA | protein coding | 8,38 | 1,1E-02 |
| ICM | (C-6) LM-HM | ALDH2 | protein coding | 2,92 | 1,4E-02 |
| ICM | (C-6) LM-HM | MTMR11 | protein coding | 9,44 | 1,5E-02 |
| ICM | (C-6) LM-HM | TRIM17 | protein coding | 5,16 | 1,5E-02 |
| ICM | (C-6) LM-HM | GYPC | protein coding | 6,34 | 1,7E-02 |
| ICM | (C-6) LM-HM | FGA | protein coding | 8,35 | 3,6E-02 |
| ICM | (C-6) LM-HM | SOX17 | protein coding | 6,02 | 3,9E-02 |
| ICM | (C-6) LM-HM | PYCR1 | protein coding | 2,17 | 4,4E-02 |
| ICM | (C-6) LM-HM | TMEM107 | protein coding | 2,18 | 4,5E-02 |
| ICM | (C-6) LM-HM | DLK2 | protein coding | -4,65 | 1,8E-10 |
| ICM | (C-6) LM-HM | GAGE2E | protein coding | -23,77 | 9,8E-07 |
| ICM | (C-6) LM-HM | GAGE12F | protein coding | -4,03 | 4,8E-06 |
| ICM | (C-6) LM-HM | LAMC3 | protein coding | -4,49 | 1,2E-05 |
| ICM | (C-6) LM-HM | PKHD1L1 | protein coding | -5,98 | 1,2E-05 |
| ICM | (C-6) LM-HM | HTR2C | protein coding | -9,28 | 2,0E-04 |
| ICM | (C-6) LM-HM | SULT4A1 | protein coding | -8,73 | 2,2E-04 |
| ICM | (C-6) LM-HM | BX842568.1 | pseudogene | -8,44 | 3,2E-04 |
| ICM | (C-6) LM-HM | CCK | protein coding | -8,84 | 8,5E-04 |
| ICM | (C-6) LM-HM | APOC4 | protein coding | -9,17 | 3,1E-03 |
| ICM | (C-6) LM-HM | RNF144B | protein coding | -6,28 | 3,2E-03 |
| ICM | (C-6) LM-HM | IL22RA1 | protein coding | -5,79 | 3,9E-03 |
| ICM | (C-6) LM-HM | PON1 | protein coding | -5,10 | 9,5E-03 |
| ICM | (C-6) LM-HM | GAGE12J | protein coding | -2,94 | 1,5E-02 |
| ICM | (C-6) LM-HM | ITIH6 | protein coding | -9,19 | 1,7E-02 |
| ICM | (C-6) LM-HM | KDM4E | protein coding | -4,39 | 1,7E-02 |
| ICM | (C-6) LM-HM | PGGHG | protein coding | -8,11 | 1,8E-02 |
| ICM | (C-6) LM-HM | C1QTNF9 | protein coding | -6,49 | 2,2E-02 |
| ICM | (C-6) LM-HM | DNASE1L1 | protein coding | -5,43 | 3,1E-02 |
| ICM | (C-6) LM-HM | AC016582.1 | pseudogene | -5,22 | 3,3E-02 |
| ICM | (C-6) LM-HM | ZNF19 | protein coding | -3,02 | 3,8E-02 |
| ICM | (C-6) LM-HM | CCL15-CCL14 | protein coding | -8,76 | 3,9E-02 |

**Supplementary Table 3. List of commonly differentially expressed genes found between euploid embryos (EU) and both low-level mosaic embryos (LM) and high-level mosaic embryos (HM).** Genes with a Benjamini-Hochberg adjusted p-value (padj) < 0.05 and a log2foldchange of ±2 was considered significantly differentially expressed. log2FoldChange > 0 indicates significant upregulation in the first comparison factor.

| **Fraction** | **Gene symbol** | **Gene biotype** | **log2FoldChange** | | **padj** | |
| --- | --- | --- | --- | --- | --- | --- |
|  |  |  | **EU-LM** | **EU-HM** | **EU-LM** | **EU-HM** |
| TE | TXLNGY | pseudogene | -11,76 | -12,20 | 3,65E-04 | 2,02E-02 |
| TE | MAGEA8 | protein coding | -6,44 | -8,25 | 2,57E-02 | 1,97E-02 |
| TE | NELL1 | protein coding | -7,86 | -8,61 | 1,81E-03 | 1,22E-06 |
| TE | NLRP13 | protein coding | -4,58 | -4,20 | 8,23E-03 | 3,79E-02 |
| TE | CPNE4 | protein coding | -4,27 | -5,57 | 1,93E-02 | 2,76E-03 |
| TE | RBMY1J | protein coding | -10,55 | -10,76 | 5,30E-06 | 4,34E-02 |
| TE | RPL7P16 | pseudogene | -7,11 | -7,35 | 1,02E-04 | 1,39E-02 |
| TE | RBMY2FP | pseudogene | -7,62 | -7,86 | 1,02E-02 | 4,06E-02 |
| TE | TSPY1 | protein coding | -8,59 | -9,24 | 8,23E-03 | 1,17E-03 |
| TE | DGKK | protein coding | 4,86 | 4,79 | 3,31E-02 | 6,72E-03 |
| ICM | SPATA20 | protein coding | 8,33 | 7,24 | 3,82E-02 | 2,83E-02 |
| ICM | FCGRT | protein coding | -5,36 | -5,31 | 1,79E-03 | 1,96E-03 |
| ICM | CNNM2 | protein coding | -2,22 | -2,37 | 3,98E-02 | 6,61E-03 |
| ICM | TRIM36 | protein coding | -7,32 | -6,53 | 1,35E-03 | 1,40E-02 |
| ICM | C10orf90 | protein coding | 8,38 | 7,01 | 5,30E-03 | 6,62E-03 |
| ICM | RBMY1J | protein coding | -9,24 | -10,68 | 4,14E-02 | 4,42E-02 |
| ICM | RPL10P9 | pseudogene | 6,35 | 7,86 | 4,78E-02 | 2,11E-03 |

**Supplementary Table 4. List of commonly differentially expressed genes found in inner cell mass (ICM) samples between high-level mosaic embryos (HM) and both low-level mosaic embryos (LM) and euploid embryos (EU).** Genes with a Benjamini-Hochberg adjusted p-value (padj) < 0.05 and a log2foldchange of ±2 was considered significantly differentially expressed. log2FoldChange > 0 indicates significant upregulation in the first comparison factor.

| **Fraction** | **Gene symbol** | **Gene biotype** | **log2FoldChange** | | **padj** | |
| --- | --- | --- | --- | --- | --- | --- |
|  |  |  | **(C-5)**  **EU-HM** | **(C-6)**  **LM-HM** | **(C-5)**  **EU-HM** | **(C-6)**  **LM-HM** |
| ICM | PLAU | protein coding | 22,33 | 9,75 | 2,46E-11 | 8,37E-05 |
| ICM | DLK2 | protein coding | -3,38 | -4,65 | 3,09E-05 | 1,76E-10 |
| ICM | CCK | protein coding | -8,54 | -8,84 | 4,12E-05 | 8,47E-04 |
| ICM | SMLR1 | protein coding | 9,37 | 9,29 | 1,03E-04 | 8,47E-04 |
| ICM | GPX2 | protein coding | 4,15 | 5,39 | 7,44E-04 | 5,74E-04 |
| ICM | ITIH6 | protein coding | -8,88 | -9,19 | 4,52E-03 | 1,65E-02 |
| ICM | ALDH2 | protein coding | 2,22 | 2,92 | 6,61E-03 | 1,40E-02 |
| ICM | CDH5 | protein coding | 8,18 | 8,31 | 6,61E-03 | 8,49E-03 |
| ICM | SOX17 | protein coding | 6,08 | 6,02 | 3,13E-02 | 3,90E-02 |
| ICM | PON1 | protein coding | -4,46 | -5,1 | 4,08E-02 | 9,51E-03 |

**Supplementary Table 5. List of exclusively differentially expressed genes found in inner cell mass (ICM) samples between euploid embryos (EU) and high-level mosaic embryos (HM).** Genes with a Benjamini-Hochberg adjusted p-value (padj) < 0.05 and a log2foldchange of ±2 was considered significantly differentially expressed. log2FoldChange > 0 indicates significant upregulation in the first comparison factor.

| **Fraction** | **Comparison** | **Gene symbol** | **Gene biotype** | **log2FoldChange** | **padj** |
| --- | --- | --- | --- | --- | --- |
| ICM | (C-5) EU-HM | VCY1B | protein coding | -27,01 | 4,80E-13 |
| ICM | (C-5) EU-HM | TRIM43 | protein coding | 22,19 | 3,55E-11 |
| ICM | (C-5) EU-HM | GAGE12C | protein coding | 24,64 | 9,64E-11 |
| ICM | (C-5) EU-HM | LRRC20 | protein coding | 21,61 | 1,75E-09 |
| ICM | (C-5) EU-HM | DRD2 | protein coding | 21,42 | 4,71E-09 |
| ICM | (C-5) EU-HM | ANXA1 | protein coding | 22,62 | 5,80E-09 |
| ICM | (C-5) EU-HM | TRIM49D1 | protein coding | 8,63 | 5,01E-07 |
| ICM | (C-5) EU-HM | IFITM1 | protein coding | 3,13 | 1,46E-05 |
| ICM | (C-5) EU-HM | TXLNGY | pseudogene | -11,89 | 4,67E-05 |
| ICM | (C-5) EU-HM | ACVR1C | protein coding | -4,83 | 7,32E-05 |
| ICM | (C-5) EU-HM | VIM | protein coding | -8,8 | 2,11E-04 |
| ICM | (C-5) EU-HM | CPXM2 | protein coding | 6,91 | 3,26E-04 |
| ICM | (C-5) EU-HM | SLC28A1 | protein coding | 7,45 | 3,26E-04 |
| ICM | (C-5) EU-HM | SLPI | protein coding | 5,58 | 9,06E-04 |
| ICM | (C-5) EU-HM | KLK13 | protein coding | 5,44 | 1,36E-03 |
| ICM | (C-5) EU-HM | GVINP1 | pseudogene | 3,67 | 1,36E-03 |
| ICM | (C-5) EU-HM | GLRX | protein coding | 3,94 | 1,69E-03 |
| ICM | (C-5) EU-HM | HNRNPA1P21 | pseudogene | 6,1 | 2,04E-03 |
| ICM | (C-5) EU-HM | MTND6P4 | pseudogene | 4,89 | 3,68E-03 |
| ICM | (C-5) EU-HM | AP000356.3 | pseudogene | 7,74 | 3,75E-03 |
| ICM | (C-5) EU-HM | MSTO2P | pseudogene | 7,59 | 3,95E-03 |
| ICM | (C-5) EU-HM | JAG1 | protein coding | -2,79 | 4,52E-03 |
| ICM | (C-5) EU-HM | MORC1 | protein coding | -8,12 | 5,35E-03 |
| ICM | (C-5) EU-HM | TSPY1 | protein coding | -8,77 | 5,35E-03 |
| ICM | (C-5) EU-HM | SEPTIN6 | protein coding | 6,61 | 6,69E-03 |
| ICM | (C-5) EU-HM | ARHGEF28 | protein coding | -6,27 | 8,34E-03 |
| ICM | (C-5) EU-HM | HNRNPA1P16 | pseudogene | 6,8 | 9,60E-03 |
| ICM | (C-5) EU-HM | GPR1 | protein coding | 2,89 | 1,01E-02 |
| ICM | (C-5) EU-HM | AC093809.1 | pseudogene | 7,58 | 1,01E-02 |
| ICM | (C-5) EU-HM | SLC6A9 | protein coding | 3,81 | 1,09E-02 |
| ICM | (C-5) EU-HM | H2BC20P | pseudogene | -4,64 | 1,55E-02 |
| ICM | (C-5) EU-HM | CXCL16 | protein coding | -2,78 | 2,15E-02 |
| ICM | (C-5) EU-HM | CAMKV | protein coding | 7,53 | 2,83E-02 |
| ICM | (C-5) EU-HM | NELL1 | protein coding | -5,25 | 3,32E-02 |
| ICM | (C-5) EU-HM | MT1H | protein coding | 3,07 | 3,32E-02 |
| ICM | (C-5) EU-HM | APOBEC3C | protein coding | 5,48 | 3,32E-02 |
| ICM | (C-5) EU-HM | PRDM11 | protein coding | 5,78 | 3,35E-02 |
| ICM | (C-5) EU-HM | PAPOLB | protein coding | 8,01 | 3,94E-02 |
| ICM | (C-5) EU-HM | NPHP3-ACAD11 | protein coding | -7,2 | 4,05E-02 |
| ICM | (C-5) EU-HM | GBA3 | pseudogene | 7,34 | 4,08E-02 |

**Supplementary Table 6. List of commonly significantly deregulated terms from functional enrichment analysis found in trophectoderm (TE) samples between euploid embryos (EU) and both low-level mosaic embryos (LM) and high-level mosaic embryos (HM).** padj: Benjamini-Hochberg adjusted p-value. KEGG: Kyoto Encyclopedia of Genes and Genomes; GO: gene ontology; BP: biological process; CC: cellular component; MF: molecular function. Normalized enrichment score (NES) > 0 indicates overrepresentation of the term in the first comparison factor.

| **Fraction** | **Category** | **Gene Set Enrichment Term** | **NES** | | **padj** | |
| --- | --- | --- | --- | --- | --- | --- |
|  |  |  | **(C1)**  **EU-LM** | **(C2)**  **EU-HM** | **(C1)**  **EU-LM** | **(C2)**  **EU-HM** |
| TE | BP | GO:0090630-activation of gtpase activity | 1,67 | 1,8 | 2,99E-02 | 1,46E-02 |
| TE | BP | GO:0019882-antigen processing and presentation | 1,5 | 1,58 | 1,90E-02 | 2,59E-02 |
| TE | BP | GO:0042590-antigen processing and presentation of exogenous peptide antigen via mhc class i | 1,62 | 1,73 | 4,03E-02 | 4,53E-02 |
| TE | BP | GO:0198738-cell cell signaling by wnt | 1,58 | 1,35 | 3,36E-04 | 3,62E-02 |
| TE | BP | GO:0044839-cell cycle g2 m phase transition | 1,8 | 1,57 | 8,91E-05 | 1,32E-02 |
| TE | BP | GO:0006884-cell volume homeostasis | 1,79 | 1,89 | 2,38E-02 | 4,37E-02 |
| TE | BP | GO:0006520-cellular amino acid metabolic process | 1,52 | 1,95 | 1,28E-02 | 1,66E-05 |
| TE | BP | GO:0042180-cellular ketone metabolic process | 1,48 | 1,64 | 4,63E-02 | 2,10E-02 |
| TE | BP | GO:0022412-cellular process involved in reproduction in multicellular organism | -1,53 | -1,51 | 6,40E-03 | 2,64E-02 |
| TE | BP | GO:0071453-cellular response to oxygen levels | 1,88 | 1,57 | 6,32E-05 | 1,18E-02 |
| TE | BP | GO:0035967-cellular response to topologically incorrect protein | 1,78 | 1,85 | 1,78E-03 | 3,58E-03 |
| TE | BP | GO:1902476-chloride transmembrane transport | -1,64 | -1,78 | 3,23E-02 | 3,71E-02 |
| TE | BP | GO:0007059-chromosome segregation | 1,5 | 1,43 | 6,75E-03 | 3,54E-02 |
| TE | BP | GO:0016569-covalent chromatin modification | 1,81 | 1,42 | 2,93E-07 | 2,13E-02 |
| TE | BP | GO:0050906-detection of stimulus involved in sensory perception | -1,61 | -1,72 | 6,62E-03 | 2,51E-03 |
| TE | BP | GO:0010256-endomembrane system organization | 1,59 | 1,73 | 5,53E-04 | 4,86E-05 |
| TE | BP | GO:0030968-endoplasmic reticulum unfolded protein response | 1,78 | 1,89 | 3,96E-03 | 4,94E-03 |
| TE | BP | GO:0036503-erad pathway | 1,66 | 1,74 | 2,16E-02 | 3,62E-02 |
| TE | BP | GO:0097191-extrinsic apoptotic signaling pathway | 1,65 | 1,63 | 1,93E-03 | 1,33E-02 |
| TE | BP | GO:0009566-fertilization | -1,55 | -1,9 | 4,95E-02 | 5,01E-04 |
| TE | BP | GO:0048144-fibroblast proliferation | 1,75 | 1,66 | 1,30E-02 | 4,53E-02 |
| TE | BP | GO:0006091-generation of precursor metabolites and energy | 1,4 | 1,65 | 1,54E-02 | 1,26E-04 |
| TE | BP | GO:0007281-germ cell development | -1,56 | -1,54 | 1,30E-02 | 3,62E-02 |
| TE | BP | GO:0007030-golgi organization | 1,68 | 1,64 | 9,88E-03 | 4,07E-02 |
| TE | BP | GO:0007249-i kappab kinase nf kappab signaling | 1,57 | 1,69 | 1,06E-02 | 5,77E-03 |
| TE | BP | GO:0097193-intrinsic apoptotic signaling pathway | 1,52 | 1,45 | 1,38E-02 | 4,83E-02 |
| TE | BP | GO:0048232-male gamete generation | -1,74 | -1,72 | 8,16E-06 | 1,95E-05 |
| TE | BP | GO:0033619-membrane protein proteolysis | 1,84 | 1,87 | 1,53E-02 | 3,35E-02 |
| TE | BP | GO:0000070-mitotic sister chromatid segregation | 1,83 | 1,79 | 6,59E-04 | 4,79E-03 |
| TE | BP | GO:0034660-ncrna metabolic process | 2,3 | 1,36 | 1,26E-07 | 4,53E-02 |
| TE | BP | GO:1902532-negative regulation of intracellular signal transduction | 1,77 | 1,51 | 1,36E-06 | 4,43E-03 |
| TE | BP | GO:0043409-negative regulation of mapk cascade | 1,9 | 1,56 | 2,29E-04 | 4,83E-02 |
| TE | BP | GO:0042326-negative regulation of phosphorylation | 1,66 | 1,38 | 2,22E-04 | 3,54E-02 |
| TE | BP | GO:0038061-nik nf kappab signaling | 1,92 | 1,77 | 3,30E-04 | 9,49E-03 |
| TE | BP | GO:0036499-perk mediated unfolded protein response | 1,86 | 2,04 | 4,99E-02 | 2,81E-02 |
| TE | BP | GO:0009896-positive regulation of catabolic process | 1,8 | 1,54 | 3,81E-06 | 4,79E-03 |
| TE | BP | GO:0043123-positive regulation of i kappab kinase nf kappab signaling | 1,68 | 1,66 | 3,60E-03 | 1,70E-02 |
| TE | BP | GO:0045732-positive regulation of protein catabolic process | 1,92 | 1,58 | 2,22E-05 | 2,87E-02 |
| TE | BP | GO:0045862-positive regulation of proteolysis | 1,78 | 1,51 | 1,34E-05 | 1,36E-02 |
| TE | BP | GO:1903052-positive regulation of proteolysis involved in cellular protein catabolic process | 1,81 | 1,61 | 2,23E-03 | 4,48E-02 |
| TE | BP | GO:1901522-positive regulation of transcription from rna polymerase ii promoter involved in cellular response to chemical stimulus | 1,95 | 1,94 | 2,83E-02 | 4,76E-02 |
| TE | BP | GO:2000060-positive regulation of ubiquitin dependent protein catabolic process | 1,86 | 1,66 | 2,78E-03 | 4,53E-02 |
| TE | BP | GO:0043687-post translational protein modification | 1,55 | 1,44 | 2,84E-03 | 3,00E-02 |
| TE | BP | GO:0010498-proteasomal protein catabolic process | 1,87 | 1,78 | 1,26E-07 | 5,28E-06 |
| TE | BP | GO:0034502-protein localization to chromosome | 2,11 | 1,81 | 2,22E-04 | 2,11E-02 |
| TE | BP | GO:0070646-protein modification by small protein removal | 1,7 | 1,44 | 4,62E-04 | 4,31E-02 |
| TE | BP | GO:0007265-ras protein signal transduction | 1,42 | 1,47 | 1,38E-02 | 1,54E-02 |
| TE | BP | GO:2001233-regulation of apoptotic signaling pathway | 1,79 | 1,61 | 1,13E-05 | 1,59E-03 |
| TE | BP | GO:1902749-regulation of cell cycle g2 m phase transition | 1,86 | 1,64 | 1,35E-04 | 1,16E-02 |
| TE | BP | GO:1903362-regulation of cellular protein catabolic process | 1,85 | 1,68 | 3,39E-05 | 4,43E-03 |
| TE | BP | GO:0045540-regulation of cholesterol biosynthetic process | 1,97 | 2,41 | 1,11E-02 | 1,06E-04 |
| TE | BP | GO:0033044-regulation of chromosome organization | 1,7 | 1,5 | 3,03E-04 | 1,72E-02 |
| TE | BP | GO:0051090-regulation of dna binding transcription factor activity | 1,44 | 1,61 | 1,30E-02 | 7,21E-04 |
| TE | BP | GO:2000278-regulation of dna biosynthetic process | 1,68 | 1,66 | 1,59E-02 | 4,12E-02 |
| TE | BP | GO:2001236-regulation of extrinsic apoptotic signaling pathway | 1,75 | 1,73 | 2,59E-03 | 1,61E-02 |
| TE | BP | GO:0046890-regulation of lipid biosynthetic process | 1,73 | 1,94 | 3,13E-03 | 4,65E-04 |
| TE | BP | GO:0019216-regulation of lipid metabolic process | 1,41 | 1,49 | 3,13E-02 | 1,70E-02 |
| TE | BP | GO:0019219-regulation of nucleobase containing compound metabolic process | 1,69 | 1,36 | 9,17E-06 | 3,62E-02 |
| TE | BP | GO:0061136-regulation of proteasomal protein catabolic process | 1,91 | 1,75 | 1,32E-04 | 4,42E-03 |
| TE | BP | GO:0032434-regulation of proteasomal ubiquitin dependent protein catabolic process | 1,94 | 1,74 | 3,70E-04 | 1,39E-02 |
| TE | BP | GO:0042176-regulation of protein catabolic process | 1,92 | 1,68 | 1,31E-07 | 5,26E-04 |
| TE | BP | GO:0071900-regulation of protein serine threonine kinase activity | 1,32 | 1,35 | 4,95E-02 | 4,53E-02 |
| TE | BP | GO:0031647-regulation of protein stability | 1,47 | 1,59 | 3,15E-02 | 6,83E-03 |
| TE | BP | GO:0010469-regulation of signaling receptor activity | -1,51 | -1,6 | 5,18E-03 | 7,26E-04 |
| TE | BP | GO:0033045-regulation of sister chromatid segregation | 1,64 | 1,8 | 3,15E-02 | 1,63E-02 |
| TE | BP | GO:0062012-regulation of small molecule metabolic process | 1,72 | 1,77 | 1,16E-04 | 1,26E-04 |
| TE | BP | GO:0050810-regulation of steroid biosynthetic process | 1,9 | 2,27 | 3,60E-03 | 4,12E-05 |
| TE | BP | GO:0019218-regulation of steroid metabolic process | 1,72 | 2,12 | 5,32E-03 | 2,57E-04 |
| TE | BP | GO:2000058-regulation of ubiquitin dependent protein catabolic process | 1,89 | 1,69 | 2,84E-04 | 2,59E-02 |
| TE | BP | GO:0030111-regulation of wnt signaling pathway | 1,62 | 1,41 | 8,07E-04 | 4,83E-02 |
| TE | BP | GO:0034976-response to endoplasmic reticulum stress | 1,68 | 1,96 | 9,68E-04 | 5,28E-06 |
| TE | BP | GO:0009408-response to heat | 1,69 | 1,62 | 3,76E-03 | 3,62E-02 |
| TE | BP | GO:0009314-response to radiation | 1,47 | 1,54 | 6,74E-03 | 2,51E-03 |
| TE | BP | GO:0035966-response to topologically incorrect protein | 1,87 | 2,06 | 3,27E-04 | 7,64E-06 |
| TE | BP | GO:0034612-response to tumor necrosis factor | 1,44 | 1,43 | 3,56E-02 | 4,92E-02 |
| TE | BP | GO:0007606-sensory perception of chemical stimulus | -1,59 | -1,76 | 5,05E-03 | 1,08E-03 |
| TE | BP | GO:0007608-sensory perception of smell | -1,54 | -1,63 | 3,14E-02 | 1,49E-02 |
| TE | BP | GO:0000819-sister chromatid segregation | 1,84 | 1,78 | 3,08E-04 | 3,60E-03 |
| TE | BP | GO:0006694-steroid biosynthetic process | 1,85 | 2,32 | 6,02E-04 | 3,83E-07 |
| TE | BP | GO:0008202-steroid metabolic process | 1,46 | 1,92 | 2,12E-02 | 5,28E-06 |
| TE | BP | GO:0016126-sterol biosynthetic process | 2,11 | 2,55 | 3,36E-04 | 9,98E-07 |
| TE | BP | GO:0032200-telomere organization | 1,71 | 1,61 | 6,21E-03 | 4,24E-02 |
| TE | BP | GO:0006099-tricarboxylic acid cycle | 2,16 | 2,09 | 1,42E-03 | 6,76E-03 |
| TE | BP | GO:0030433-ubiquitin dependent erad pathway | 1,68 | 1,78 | 4,95E-02 | 2,87E-02 |
| TE | BP | GO:0019058-viral life cycle | 1,69 | 1,5 | 3,18E-04 | 1,63E-02 |
| TE | CC | GO:0015629-actin cytoskeleton | 1,26 | 1,41 | 3,92E-02 | 1,27E-02 |
| TE | CC | GO:0101031-chaperone complex | 2,12 | 1,89 | 3,63E-03 | 4,93E-02 |
| TE | CC | GO:0034707-chloride channel complex | -1,78 | -1,77 | 1,60E-02 | 4,45E-02 |
| TE | CC | GO:0098687-chromosomal region | 1,73 | 1,62 | 6,83E-05 | 4,48E-03 |
| TE | CC | GO:0000775-chromosome centromeric region | 1,42 | 1,51 | 4,60E-02 | 3,21E-02 |
| TE | CC | GO:0097014-ciliary plasm | -1,7 | -1,64 | 9,60E-03 | 3,98E-02 |
| TE | CC | GO:0031461-cullin ring ubiquitin ligase complex | 1,8 | 1,66 | 8,13E-04 | 1,51E-02 |
| TE | CC | GO:0031045-dense core granule | -1,99 | -2,09 | 5,76E-03 | 8,01E-03 |
| TE | CC | GO:1905369-endopeptidase complex | 1,6 | 1,85 | 2,54E-02 | 1,76E-02 |
| TE | CC | GO:0044440-endosomal part | 1,72 | 1,5 | 1,78E-06 | 4,48E-03 |
| TE | CC | GO:0101002-ficolin 1 rich granule | 1,69 | 1,92 | 7,00E-03 | 2,91E-03 |
| TE | CC | GO:0031300-intrinsic component of organelle membrane | 1,43 | 1,56 | 1,79E-02 | 4,21E-03 |
| TE | CC | GO:0000776-kinetochore | 1,43 | 1,53 | 4,40E-02 | 4,33E-02 |
| TE | CC | GO:0005770-late endosome | 1,61 | 1,64 | 3,70E-03 | 4,72E-03 |
| TE | CC | GO:0031902-late endosome membrane | 1,66 | 1,57 | 9,95E-03 | 3,48E-02 |
| TE | CC | GO:0043202-lysosomal lumen | 1,54 | 1,85 | 3,29E-02 | 8,56E-03 |
| TE | CC | GO:0005759-mitochondrial matrix | 1,77 | 1,85 | 1,20E-06 | 1,31E-06 |
| TE | CC | GO:0005635-nuclear envelope | 1,59 | 1,46 | 9,24E-05 | 1,20E-02 |
| TE | CC | GO:0034399-nuclear periphery | 2,04 | 1,55 | 2,21E-05 | 4,33E-02 |
| TE | CC | GO:0016607-nuclear speck | 1,7 | 1,37 | 4,45E-05 | 3,98E-02 |
| TE | CC | GO:0055037-recycling endosome | 1,5 | 1,6 | 2,66E-02 | 1,76E-02 |
| TE | CC | GO:0005819-spindle | 1,51 | 1,46 | 4,24E-03 | 1,76E-02 |
| TE | CC | GO:1990351-transporter complex | -1,47 | -1,5 | 1,55E-02 | 1,81E-02 |
| TE | CC | GO:0000151-ubiquitin ligase complex | 1,75 | 1,56 | 6,41E-05 | 9,71E-03 |
| TE | CC | GO:0005775-vacuolar lumen | 1,48 | 1,53 | 2,33E-02 | 4,53E-02 |
| TE | CC | GO:0005774-vacuolar membrane | 1,79 | 1,53 | 3,05E-06 | 4,48E-03 |
| TE | CC | GO:0031983-vesicle lumen | 1,41 | 1,61 | 1,88E-02 | 4,48E-03 |
| TE | MF | GO:0005254-chloride channel activity | -1,64 | -1,75 | 2,64E-02 | 3,19E-02 |
| TE | MF | GO:0005230-extracellular ligand gated ion channel activity | -1,76 | -1,82 | 1,18E-02 | 1,62E-02 |
| TE | MF | GO:0030594-neurotransmitter receptor activity | -1,74 | -1,66 | 7,44E-03 | 3,89E-02 |
| TE | MF | GO:0004984-olfactory receptor activity | -1,6 | -1,54 | 1,16E-02 | 2,61E-02 |
| TE | MF | GO:0022803-passive transmembrane transporter activity | -1,33 | -1,45 | 4,51E-02 | 2,43E-02 |
| TE | MF | GO:0030545-receptor regulator activity | -1,53 | -1,6 | 2,58E-03 | 1,25E-03 |
| TE | MF | GO:0022835-transmitter gated channel activity | -2,03 | -2,1 | 1,59E-03 | 1,25E-03 |
| TE | KEGG | 00020_KEGG-citrate cycle tca cycle | 2,39 | 2,26 | 1,85E-05 | 5,74E-04 |
| TE | KEGG | 11266_KEGG-lysosome | 1,93 | 1,81 | 5,32E-04 | 4,98E-03 |
| TE | KEGG | 13380_KEGG-neuroactive ligand receptor interaction | -1,66 | -1,53 | 2,39E-03 | 1,80E-02 |
| TE | KEGG | 5872_KEGG-steroid biosynthesis | 2,02 | 2,32 | 1,28E-02 | 4,47E-04 |
| TE | KEGG | 19428_KEGG-wnt signaling pathway | 1,59 | 1,49 | 2,12E-02 | 4,34E-02 |

**Supplementary Table 7. List of significantly deregulated terms from functional enrichment analysis found in inner cell mass (ICM) samples between low-level mosaic embryos (LM) and high-level mosaic embryos (HM).** padj: Benjamini-Hochberg adjusted p-value. KEGG: Kyoto Encyclopedia of Genes and Genomes; GO: gene ontology; BP: biological process; CC: cellular component; MF: molecular function. Normalized enrichment score (NES) > 0 indicates overrepresentation of the term in the first comparison factor.

| **Fraction** | **Comparison** | **Category** | **Gene Set Enrichment Term** | **NES** | **padj** |
| --- | --- | --- | --- | --- | --- |
| ICM | C6 (LM-HM) | BP | GO:0006613-cotranslational protein targeting to membrane | 2,73 | 1,28E-07 |
| ICM | C6 (LM-HM) | BP | GO:0072599-establishment of protein localization to endoplasmic reticulum | 2,6 | 1,28E-07 |
| ICM | C6 (LM-HM) | BP | GO:0006612-protein targeting to membrane | 2,34 | 1,28E-07 |
| ICM | C6 (LM-HM) | BP | GO:0000184-nuclear transcribed mrna catabolic process nonsense mediated decay | 2,34 | 3,12E-07 |
| ICM | C6 (LM-HM) | BP | GO:0070972-protein localization to endoplasmic reticulum | 2,31 | 3,12E-07 |
| ICM | C6 (LM-HM) | BP | GO:0006605-protein targeting | 1,86 | 5,90E-07 |
| ICM | C6 (LM-HM) | BP | GO:0090150-establishment of protein localization to membrane | 1,96 | 7,66E-07 |
| ICM | C6 (LM-HM) | BP | GO:0015980-energy derivation by oxidation of organic compounds | 1,86 | 1,03E-04 |
| ICM | C6 (LM-HM) | BP | GO:0001655-urogenital system development | 1,81 | 1,03E-04 |
| ICM | C6 (LM-HM) | BP | GO:0006091-generation of precursor metabolites and energy | 1,55 | 7,86E-04 |
| ICM | C6 (LM-HM) | BP | GO:0022409-positive regulation of cell cell adhesion | 1,8 | 7,86E-04 |
| ICM | C6 (LM-HM) | BP | GO:0060602-branch elongation of an epithelium | 2,3 | 1,02E-03 |
| ICM | C6 (LM-HM) | BP | GO:0045333-cellular respiration | 1,79 | 1,92E-03 |
| ICM | C6 (LM-HM) | BP | GO:0072001-renal system development | 1,7 | 1,92E-03 |
| ICM | C6 (LM-HM) | BP | GO:0006413-translational initiation | 1,79 | 1,92E-03 |
| ICM | C6 (LM-HM) | BP | GO:2001233-regulation of apoptotic signaling pathway | 1,6 | 2,25E-03 |
| ICM | C6 (LM-HM) | BP | GO:0019080-viral gene expression | 1,77 | 2,29E-03 |
| ICM | C6 (LM-HM) | BP | GO:0022407-regulation of cell cell adhesion | 1,6 | 3,34E-03 |
| ICM | C6 (LM-HM) | BP | GO:0003401-axis elongation | 2,24 | 3,92E-03 |
| ICM | C6 (LM-HM) | BP | GO:0051186-cofactor metabolic process | 1,52 | 4,63E-03 |
| ICM | C6 (LM-HM) | BP | GO:0061326-renal tubule development | 1,97 | 5,33E-03 |
| ICM | C6 (LM-HM) | BP | GO:0000956-nuclear transcribed mrna catabolic process | 1,71 | 6,28E-03 |
| ICM | C6 (LM-HM) | BP | GO:0001667-ameboidal type cell migration | 1,53 | 6,96E-03 |
| ICM | C6 (LM-HM) | BP | GO:0050727-regulation of inflammatory response | 1,62 | 6,96E-03 |
| ICM | C6 (LM-HM) | BP | GO:0001763-morphogenesis of a branching structure | 1,72 | 7,09E-03 |
| ICM | C6 (LM-HM) | BP | GO:0050821-protein stabilization | 1,72 | 8,19E-03 |
| ICM | C6 (LM-HM) | BP | GO:1901605-alpha amino acid metabolic process | 1,7 | 1,04E-02 |
| ICM | C6 (LM-HM) | BP | GO:0050673-epithelial cell proliferation | 1,52 | 1,21E-02 |
| ICM | C6 (LM-HM) | BP | GO:0045785-positive regulation of cell adhesion | 1,52 | 1,21E-02 |
| ICM | C6 (LM-HM) | BP | GO:0052547-regulation of peptidase activity | 1,5 | 1,37E-02 |
| ICM | C6 (LM-HM) | BP | GO:0072028-nephron morphogenesis | 1,89 | 1,38E-02 |
| ICM | C6 (LM-HM) | BP | GO:0048754-branching morphogenesis of an epithelial tube | 1,75 | 1,54E-02 |
| ICM | C6 (LM-HM) | BP | GO:0022900-electron transport chain | 1,67 | 1,54E-02 |
| ICM | C6 (LM-HM) | BP | GO:0007369-gastrulation | 1,65 | 1,54E-02 |
| ICM | C6 (LM-HM) | BP | GO:0090103-cochlea morphogenesis | 2,08 | 1,65E-02 |
| ICM | C6 (LM-HM) | BP | GO:0030850-prostate gland development | 2,03 | 1,65E-02 |
| ICM | C6 (LM-HM) | BP | GO:0019216-regulation of lipid metabolic process | 1,49 | 1,78E-02 |
| ICM | C6 (LM-HM) | BP | GO:0061053-somite development | 1,9 | 1,78E-02 |
| ICM | C6 (LM-HM) | BP | GO:0045834-positive regulation of lipid metabolic process | 1,71 | 1,79E-02 |
| ICM | C6 (LM-HM) | BP | GO:0072079-nephron tubule formation | 2,05 | 1,89E-02 |
| ICM | C6 (LM-HM) | BP | GO:0016054-organic acid catabolic process | 1,55 | 1,89E-02 |
| ICM | C6 (LM-HM) | BP | GO:0007156-homophilic cell adhesion via plasma membrane adhesion molecules | -1,75 | 1,90E-02 |
| ICM | C6 (LM-HM) | BP | GO:0060192-negative regulation of lipase activity | 2,04 | 2,06E-02 |
| ICM | C6 (LM-HM) | BP | GO:0034340-response to type i interferon | 1,83 | 2,28E-02 |
| ICM | C6 (LM-HM) | BP | GO:0060512-prostate gland morphogenesis | 2 | 2,96E-02 |
| ICM | C6 (LM-HM) | BP | GO:0098754-detoxification | 1,75 | 3,15E-02 |
| ICM | C6 (LM-HM) | BP | GO:0072171-mesonephric tubule morphogenesis | 1,87 | 3,30E-02 |
| ICM | C6 (LM-HM) | BP | GO:0042060-wound healing | 1,37 | 3,30E-02 |
| ICM | C6 (LM-HM) | BP | GO:0043434-response to peptide hormone | 1,43 | 3,34E-02 |
| ICM | C6 (LM-HM) | BP | GO:0048546-digestive tract morphogenesis | 1,91 | 3,34E-02 |
| ICM | C6 (LM-HM) | BP | GO:0048732-gland development | 1,39 | 3,34E-02 |
| ICM | C6 (LM-HM) | BP | GO:1904035-regulation of epithelial cell apoptotic process | 1,82 | 3,34E-02 |
| ICM | C6 (LM-HM) | BP | GO:0030178-negative regulation of wnt signaling pathway | 1,55 | 3,35E-02 |
| ICM | C6 (LM-HM) | BP | GO:0006119-oxidative phosphorylation | 1,67 | 3,35E-02 |
| ICM | C6 (LM-HM) | BP | GO:0009617-response to bacterium | 1,36 | 3,35E-02 |
| ICM | C6 (LM-HM) | BP | GO:0035270-endocrine system development | 1,7 | 3,35E-02 |
| ICM | C6 (LM-HM) | BP | GO:0009636-response to toxic substance | 1,38 | 3,35E-02 |
| ICM | C6 (LM-HM) | BP | GO:0048645-animal organ formation | 1,84 | 3,42E-02 |
| ICM | C6 (LM-HM) | BP | GO:0070555-response to interleukin 1 | 1,55 | 3,42E-02 |
| ICM | C6 (LM-HM) | BP | GO:0019731-antibacterial humoral response | 1,98 | 3,51E-02 |
| ICM | C6 (LM-HM) | BP | GO:0060914-heart formation | 1,96 | 3,51E-02 |
| ICM | C6 (LM-HM) | BP | GO:0002526-acute inflammatory response | 1,78 | 3,52E-02 |
| ICM | C6 (LM-HM) | BP | GO:0031016-pancreas development | 1,77 | 3,52E-02 |
| ICM | C6 (LM-HM) | BP | GO:1903039-positive regulation of leukocyte cell cell adhesion | 1,56 | 3,52E-02 |
| ICM | C6 (LM-HM) | BP | GO:0090102-cochlea development | 1,87 | 3,62E-02 |
| ICM | C6 (LM-HM) | BP | GO:0006401-rna catabolic process | 1,43 | 3,62E-02 |
| ICM | C6 (LM-HM) | BP | GO:0051604-protein maturation | 1,52 | 3,65E-02 |
| ICM | C6 (LM-HM) | BP | GO:0009066-aspartate family amino acid metabolic process | 1,89 | 3,67E-02 |
| ICM | C6 (LM-HM) | BP | GO:0031338-regulation of vesicle fusion | 1,97 | 3,75E-02 |
| ICM | C6 (LM-HM) | BP | GO:0035357-peroxisome proliferator activated receptor signaling pathway | 1,95 | 3,78E-02 |
| ICM | C6 (LM-HM) | BP | GO:0001704-formation of primary germ layer | 1,65 | 3,80E-02 |
| ICM | C6 (LM-HM) | BP | GO:0060562-epithelial tube morphogenesis | 1,5 | 3,82E-02 |
| ICM | C6 (LM-HM) | BP | GO:0009060-aerobic respiration | 1,77 | 3,91E-02 |
| ICM | C6 (LM-HM) | BP | GO:0043062-extracellular structure organization | 1,41 | 3,94E-02 |
| ICM | C6 (LM-HM) | BP | GO:0032355-response to estradiol | 1,66 | 3,94E-02 |
| ICM | C6 (LM-HM) | BP | GO:0031018-endocrine pancreas development | 1,89 | 3,96E-02 |
| ICM | C6 (LM-HM) | BP | GO:0007492-endoderm development | 1,74 | 3,96E-02 |
| ICM | C6 (LM-HM) | BP | GO:0071887-leukocyte apoptotic process | 1,73 | 3,96E-02 |
| ICM | C6 (LM-HM) | BP | GO:0031647-regulation of protein stability | 1,5 | 4,11E-02 |
| ICM | C6 (LM-HM) | BP | GO:0042773-atp synthesis coupled electron transport | 1,69 | 4,12E-02 |
| ICM | C6 (LM-HM) | BP | GO:0006520-cellular amino acid metabolic process | 1,5 | 4,21E-02 |
| ICM | C6 (LM-HM) | BP | GO:0071542-dopaminergic neuron differentiation | 1,9 | 4,21E-02 |
| ICM | C6 (LM-HM) | BP | GO:0051154-negative regulation of striated muscle cell differentiation | 1,95 | 4,21E-02 |
| ICM | C6 (LM-HM) | BP | GO:0051051-negative regulation of transport | 1,39 | 4,21E-02 |
| ICM | C6 (LM-HM) | BP | GO:0016485-protein processing | 1,57 | 4,21E-02 |
| ICM | C6 (LM-HM) | BP | GO:0010632-regulation of epithelial cell migration | 1,53 | 4,21E-02 |
| ICM | C6 (LM-HM) | BP | GO:0009069-serine family amino acid metabolic process | 1,92 | 4,21E-02 |
| ICM | C6 (LM-HM) | BP | GO:0042398-cellular modified amino acid biosynthetic process | 1,9 | 4,57E-02 |
| ICM | C6 (LM-HM) | BP | GO:0060993-kidney morphogenesis | 1,75 | 4,57E-02 |
| ICM | C6 (LM-HM) | BP | GO:0072009-nephron epithelium development | 1,64 | 4,57E-02 |
| ICM | C6 (LM-HM) | BP | GO:2000108-positive regulation of leukocyte apoptotic process | 1,85 | 4,57E-02 |
| ICM | C6 (LM-HM) | BP | GO:0043542-endothelial cell migration | 1,48 | 4,60E-02 |
| ICM | C6 (LM-HM) | BP | GO:0006635-fatty acid beta oxidation | 1,72 | 4,60E-02 |
| ICM | C6 (LM-HM) | BP | GO:0001823-mesonephros development | 1,72 | 4,60E-02 |
| ICM | C6 (LM-HM) | BP | GO:0090130-tissue migration | 1,47 | 4,60E-02 |
| ICM | C6 (LM-HM) | BP | GO:0034116-positive regulation of heterotypic cell cell adhesion | 1,94 | 4,83E-02 |
| ICM | C6 (LM-HM) | BP | GO:0009142-nucleoside triphosphate biosynthetic process | 1,71 | 4,89E-02 |
| ICM | C6 (LM-HM) | BP | GO:0032869-cellular response to insulin stimulus | 1,53 | 4,90E-02 |
| ICM | C6 (LM-HM) | CC | GO:0022626-cytosolic ribosome | 2,53 | 2,15E-08 |
| ICM | C6 (LM-HM) | CC | GO:0044391-ribosomal subunit | 2,33 | 2,15E-08 |
| ICM | C6 (LM-HM) | CC | GO:0005840-ribosome | 2,13 | 2,15E-08 |
| ICM | C6 (LM-HM) | CC | GO:0015934-large ribosomal subunit | 2,18 | 5,94E-06 |
| ICM | C6 (LM-HM) | CC | GO:0022625-cytosolic large ribosomal subunit | 2,38 | 8,43E-06 |
| ICM | C6 (LM-HM) | CC | GO:0044455-mitochondrial membrane part | 1,72 | 5,61E-04 |
| ICM | C6 (LM-HM) | CC | GO:0098798-mitochondrial protein complex | 1,71 | 5,61E-04 |
| ICM | C6 (LM-HM) | CC | GO:0044445-cytosolic part | 1,67 | 2,18E-03 |
| ICM | C6 (LM-HM) | CC | GO:0098800-inner mitochondrial membrane protein complex | 1,78 | 2,22E-03 |
| ICM | C6 (LM-HM) | CC | GO:0015935-small ribosomal subunit | 2 | 2,22E-03 |
| ICM | C6 (LM-HM) | CC | GO:0005788-endoplasmic reticulum lumen | 1,58 | 3,06E-03 |
| ICM | C6 (LM-HM) | CC | GO:0022627-cytosolic small ribosomal subunit | 2,04 | 3,88E-03 |
| ICM | C6 (LM-HM) | CC | GO:0031983-vesicle lumen | 1,56 | 3,88E-03 |
| ICM | C6 (LM-HM) | CC | GO:0043194-axon initial segment | -1,92 | 2,57E-02 |
| ICM | C6 (LM-HM) | CC | GO:0043235-receptor complex | -1,51 | 2,57E-02 |
| ICM | C6 (LM-HM) | CC | GO:0070469-respirasome | 1,68 | 2,57E-02 |
| ICM | C6 (LM-HM) | CC | GO:0098803-respiratory chain complex | 1,71 | 3,60E-02 |
| ICM | C6 (LM-HM) | CC | GO:0005759-mitochondrial matrix | 1,36 | 4,25E-02 |
| ICM | C6 (LM-HM) | CC | GO:0030867-rough endoplasmic reticulum membrane | 1,84 | 4,54E-02 |
| ICM | C6 (LM-HM) | MF | GO:0003735-structural constituent of ribosome | 2,54 | 7,46E-08 |
| ICM | C6 (LM-HM) | MF | GO:0051082-unfolded protein binding | 1,89 | 8,46E-03 |
| ICM | C6 (LM-HM) | MF | GO:0009055-electron transfer activity | 1,84 | 2,32E-02 |
| ICM | C6 (LM-HM) | KEGG | KEGG_03010-ribosome | 2,66 | 1,74E-08 |
| ICM | C6 (LM-HM) | KEGG | KEGG_00190-oxidative phosphorylation | 1,98 | 1,09E-04 |
| ICM | C6 (LM-HM) | KEGG | KEGG_05012-parkinsons disease | 1,91 | 5,06E-04 |
| ICM | C6 (LM-HM) | KEGG | KEGG_05016-huntingtons disease | 1,71 | 5,24E-03 |
| ICM | C6 (LM-HM) | KEGG | KEGG_05010-alzheimers disease | 1,73 | 6,41E-03 |
| ICM | C6 (LM-HM) | KEGG | KEGG_00330-arginine and proline metabolism | 1,93 | 7,38E-03 |
| ICM | C6 (LM-HM) | KEGG | KEGG_03320-ppar signaling pathway | 1,75 | 1,93E-02 |
| ICM | C6 (LM-HM) | KEGG | KEGG_05200-pathways in cancer | 1,42 | 2,28E-02 |
| ICM | C6 (LM-HM) | KEGG | KEGG_00310-lysine degradation | 1,82 | 2,86E-02 |
| ICM | C6 (LM-HM) | KEGG | KEGG_00650-butanoate metabolism | 1,85 | 3,65E-02 |
| ICM | C6 (LM-HM) | KEGG | KEGG_05215-prostate cancer | 1,63 | 3,65E-02 |
| ICM | C6 (LM-HM) | KEGG | KEGG_00620-pyruvate metabolism | 1,74 | 3,94E-02 |
| ICM | C6 (LM-HM) | KEGG | KEGG_00563-glycosylphosphatidylinositol gpi anchor biosynthesis | -1,79 | 4,54E-02 |
| ICM | C6 (LM-HM) | KEGG | KEGG_00260-glycine serine and threonine metabolism | 1,8 | 4,62E-02 |
